# Impact of hypertension prevalence trend on mortality and burdens of dementia and disability in England and Wales to 2060: a simulation modelling study

**DOI:** 10.1016/S2666-7568(23)00129-0

**Published:** 2023-09

**Authors:** Yuntao Chen, Marzieh Araghi, Piotr Bandosz, Martin J Shipley, Sara Ahmadi-Abhari, Sophia Lobanov-Rostovsky, Tishya Venkatraman, Mika Kivimaki, Martin O'Flaherty, Eric J Brunner

**Affiliations:** a Department of Epidemiology and Public Health, University College London, London, UK; b Department of Public Health and Policy, University of Liverpool, Liverpool, UK; c Division of Prevention Medicine & Education, Medical University of Gdansk, Gdansk, Poland; d Ageing Epidemiology Research Unit, School of Public Health, Imperial College London, London, UK

## Abstract

**Background:**

Previous estimates of the impact of public health interventions targeting hypertension usually focus on one health outcome. This study aims to consider the effects of change in future hypertension prevalence on mortality, dementia, and disability simultaneously.

**Methods:**

We modelled three plausible scenarios based on observed trends of hypertension prevalence from 2003 to 2017 in England: observed trends continue (baseline scenario); 2017 prevalence remains unchanged; and 2017 prevalence decreases by 50% by 2060. We used a probabilistic Markov model to integrate calendar trends in incidence of cardiovascular disease, dementia, disability, and mortality to forecast their future occurrence in the population of England and Wales. Assuming the hypertension prevalence trend modifies health transition probabilities, we compared mortality outcomes and the burden of dementia and disability to 2060 for the scenarios.

**Findings:**

If the decline in hypertension prevalence stops, there would be a slight increase in the number of additional deaths to 2060 (22·9 [95% uncertainty interval 19·0–26·6] more deaths per 100 000 population), although the burdens of disability and dementia in absolute terms would change little. Alternatively, if the downward hypertension prevalence trend accelerates (with prevalence falling by 50% between 2017 and 2060), there would be a modest additional reduction in deaths (57·0 [50·4–63·5] fewer deaths per 100 000 population), a small increase in dementia burden (9·0 [5·1–13·2] more cases per 100 000 population), no significant effect on disability burden, and an 8% gain in healthy life expectancy at age 65 years from 2020 to 2060 (5·3 years *vs* 4·9 years) compared with the baseline scenario.

**Interpretation:**

The major future impact of alternative hypertension prevention strategies appears to be on future life expectancy. The salutary effect of lower population blood pressure distribution on incidence of dementia and disability might not offset expansion of the susceptible population due to reduced mortality.

**Funding:**

British Heart Foundation and UK Economic and Social Research Council.

## Introduction

Dementia is a growing, global challenge, with the number of people living with this condition expected to increase by 57% between 2016 and 2040 in England and Wales.^1^ Mechanistic and therapeutic research^2^ has, to date, been unsuccessful in providing a cure for Alzheimer's disease and related dementias. However, the decades-long preclinical phase provides a window of opportunity for prevention.^3^, ^4^, ^5^, ^6^, ^7^

Hypertension is highly prevalent in midlife, and frequently not optimally treated or controlled, emphasising the importance of population initiatives to reduce blood pressure.^8^ The adverse effect of hypertension on cognitive function was first characterised in the 1960s.^9^ In recent years, increasing evidence suggests that midlife hypertension is an important risk factor for dementia.^4^, ^10^, ^11^, ^12^ Excessive salt intake is a major risk factor for high blood pressure^13^ and reduced intake would substantially reduce this risk.^14^ Awareness campaigns, salt-reduction target setting, voluntary product reformulation, and population monitoring of salt consumption^15^ are common policy approaches that have played important roles in reducing hypertension prevalence.^16^

The main health goal of hypertension reduction campaigns is to reduce the burden of cardiovascular disease (CVD); however, in parallel, there will be effects on dementia and disability incidence and prevalence.^3^, ^4^, ^10^, ^11^ If hypertension-related mortality falls, there might be, paradoxically, an increase in the burden of dementia and disability because the number of older people susceptible to dementia grows. Simultaneous modelling of demographic trends, and the trends in CVD, dementia, and mortality occurrence allows estimation of the potential effect of continuing primary prevention of hypertension on dementia and disability occurrence. Also, it allows a more realistic projection of healthy life-years (free of CVD, dementia, and disability) gained from the hypertension prevention. In this study, we used a validated Markov model, IMPACT-Better Ageing Model (BAM),^1^, ^17^, ^18^ that integrates calendar trends in incidence of CVD, dementia, disability, and mortality, to estimate the potential effects of the future trend in hypertension prevalence on mortality outcomes and the burden of dementia and disability in England and Wales to 2060.

**Research in context**

**Evidence before this study**

We searched PubMed for any studies published from inception until May 20, 2023, that estimated the impact of the hypertension prevalence trend on mortality and future burdens of disability and dementia in the UK, with the search terms “disability”, “dementia”, “longevity”, “life expectancy”, “hypertension”, “simulation”, and synonyms of “United Kingdom”. We did additional hand searches with lists of references retrieved from relevant papers. We found three studies estimating mortality and life-years gained from hypertension or salt reduction strategies in the UK. We found one study estimating the effect of hypertension prevention on future dementia burden, but it did not consider that hypertension prevention strategies could increase time spent living with dementia due to consequent mortality rate reduction. We found no studies estimating the impact of projected trends in hypertension prevalence on future disability burden.

**Added value of this study**

We estimated the impact of the hypertension prevalence trend on mortality and future burdens of dementia and disability in England and Wales, taking into account the effect of hypertension prevention strategies on dementia, disability, and mortality simultaneously. We found that the major future impact of alternative hypertension prevention strategies appears to be on total and healthy life expectancy. The effect at population level of different hypertension control policies on dementia and disability occurrence is modest. Our study suggests, perhaps counterintuitively, that the salutary effect of lower population blood pressure distribution on incidence of dementia and disability might not offset expansion of the susceptible population due to reduced mortality.

**Implications of all the available evidence**

Midlife hypertension is a shared risk factor for dementia, cardiovascular disease, and death. Therefore, in order to more accurately forecast the future burden of dementia and disability, modelling studies need to consider the effect of prevention strategies on these conditions simultaneously. By doing so, our study suggests public health interventions aimed at decreasing the incidence of hypertension might contribute to increasing healthy life expectancy in the future, but might not decrease the future burden of dementia and disability. Further research is needed to estimate the future cost and cost-effectiveness of alternative hypertension prevention strategies.

## Methods

### Case definitions

Disability was defined as the inability to independently carry out one or more activities of daily living, which included getting in or out of bed, walking across a room, bathing or showering, using the toilet, dressing, cutting food, and eating.^19^ This definition of disability captures individuals who have difficulty maintaining independence and require supportive care.

Cognitive impairment was defined as an impairment in two or more functional tests (such as orientation to time, immediate and delayed memory, verbal fluency, and numeracy function) or a score higher than 3·6 on the Informant Questionnaire on Cognitive Decline in the Elderly (IQCODE).^18^

Dementia was defined based on the coexistence of cognitive impairment and disability, or a report of a doctor diagnosis of dementia by the participant or caregiver. We adapted the case definition of dementia to resemble DSM-IV and other criteria (such as NINDES-AIREN^20^ and NINCDS-ADRDA^21^) for diagnosis of dementia.

### Overview of the IMPACT-BAM model

IMPACT-BAM^1^, ^17^ is a probabilistic Markov model that tracks the progression of the population aged 35–100 years in England and Wales from 2006 onwards through ten health states characterised by the presence or absence of CVD, cognitive impairment, dementia, disability, or death (appendix p 3). At baseline (year 2006), each health state in the model is populated using the population estimates from the Office for National Statistics (ONS) and prevalence of the above conditions from the English Longitudinal Study of Ageing (ELSA). At each iteration of the model, individuals move between the eight health states and from health states to death, governed by 1-year transition probabilities. A new cohort of 35-year-olds enters the simulation through the disease-free state. Numbers of men and women who reach age 35 years and enter the model at each calendar year are obtained from ONS population projections. These principal projections are based on assumptions regarding future levels of fertility, mortality, and migration.^22^ Combined data from ELSA and mortality projections were used to calculate the transition probabilities. Projections to 2060 were conducted separately for CVD and non-CVD mortality rates based on WHO mortality data. Based on observed mortality data for 2007–16, we projected trends for mortality rates in each 5-year age by sex group using Poisson regression assuming a log-linear association between calendar year and mortality. Detailed mathematical representation and underlying assumptions of IMPACT-BAM are provided in the appendix (pp 2–19).

### Future trends in hypertension prevalence

We modelled three plausible future hypertension prevalence scenarios based on observed trends of hypertension prevalence in the Health Survey for England.^23^ Hypertension was defined as uncontrolled or untreated systolic blood pressure of 140 mm Hg or higher, or diastolic blood pressure of 90 mm Hg or higher, for the period of 2003–17 for men and women aged 35 years and older, stratified into 5-year age groups.

We examined three scenarios, based on current trends in age and sex specific prevalence of hypertension (appendix p 23) as follows: (1) baseline: continuing trend (on the logarithmic scale) of observed trends in prevalence 2003–17; (2) constant: hypertension prevalence at the last observed year (2017) remains constant; (3) optimistic: hypertension prevalence in 2017 would be reduced by 50% by 2060.

### Effect of hypertension trends on transition probabilities

We assumed that trends in the prevalence of hypertension in each scenario would modify the relevant IMPACT-BAM transition probabilities, resulting in changes in the burden of dementia and disability. The affected transition probabilities were those representing risk of CVD and non-CVD death, incidence of CVD, cognitive impairment, disability, and recovery from disability. To model these changes in transition probabilities, we used an approach based on the population attributable risk fraction (PARF). PARF calculates the proportion by which disease burden would be reduced if hypertension prevalence was reduced to zero. We used Levin's formula to calculate PARF:^24^

$$\text{PARF}=\frac{\text{P}\times (\text{RR}-1)}{1+\text{P}\times (\text{RR}-1)}$$

where P is the hypertension prevalence, and RR is the relative risk comparing the exposure group with the unexposed group. P and RR are age and sex specific. In this study, we are interested in how PARF varies because of changes in hypertension prevalence, as per the following formula:

ΔPARF=(P-P')×(RR-1)1+P×(RR-1)

where Pʹ is the hypertension prevalence after the intervention. This equation is equivalent to the potential impact fraction (PIF) for discrete risk factors.

We took adjusted RRs that are controlled for other potential confounders from published meta-analyses and longitudinal studies (appendix p 27).

### Outcomes

For each scenario, the ΔPARF was then multiplied by the appropriate transition probability to generate a new transition probability. We repeated this procedure for all the transition probabilities. We then recalculated the IMPACT model with the new set of transition probabilities and generated scenario-specific numbers of cases of dementia, disability, and deaths from 2016 onwards and compared them against the baseline scenario (ie, the model with the original transition probability values).

We report the cumulative number of new cases of dementia, disability, and deaths attributable to each scenario for the population aged 65 years and older, in comparison with the baseline scenario. We also present life-years gained and total and healthy life expectancy (healthy refers to no CVD, dementia, or disability) at age 65 years for each scenario.

Parameter uncertainty was built into the Markov model outputs using a probabilistic approach with Monte Carlo simulation. The procedure entailed iterative sampling from specified distributions of the model input parameters to recalculate the outputs. We performed 1000 iterations to estimate 95% uncertainty intervals for the output variables.

### Sensitivity analysis

We considered the observed stalling progress in life expectancy in the UK after 2010 (appendix p 25). We used 7-year (2010–16) instead of 10-year (2007–16) observed mortality data to predict future mortality rates. Furthermore, six additional mortality trend scenarios (most pessimistically from no improvement in mortality rate to, optimistically, a doubling in the declining mortality rate trend compared with that in 2010–16) were modelled to estimate future dementia and disability trends.

The model was developed as a package in R software (version 3.4.2).

### Role of the funding source

The funders of the study had no role in study design, data collection, data analysis, data interpretation, or writing of the report.

## Results

Assuming recent trends in hypertension prevalence continue to 2060 (table 1), the number of incident disability cases among the population aged 65 years and older is predicted to decrease marginally from 2020 to 2040, and then to decline faster to 2060. The number of incident dementia cases is predicted to increase slightly from approximately 104 000 in 2020 to 107 000 in 2040 and then to fall to 88 000 in 2060.

**Table 1:** Projected number of deaths and mortality rate and incident cases of disability and dementia in the England and Wales population aged ≥65 years in 2020, 2040, and 2060 for the baseline hypertension scenario

	**Disability incident cases**		**Dementia incident cases**		**Total deaths**	
	Number (thousands)	Cases per 1000 population	Number (thousands)	Cases per 1000 population	Number (thousands)	Cases per 1000 population
**All**						
2020	163 (159–166)	18·3 (17·9–18·7)	104 (100–107)	11·6 (11·2–12·1)	485 (483–487)	54·5 (54·2–54·8)
2040	158 (153–162)	13·4 (12·9–13·8)	107 (102–111)	9·0 (8·6–9·5)	540 (539–541)	45·6 (45·4–45·9)
2060	126 (121–130)	9·0 (8·6–9·4)	88 (83–93)	6·3 (5·9–6·7)	598 (596–600)	42·7 (42·6–42·8)
**Men**						
2020	71 (68–74)	18·1 (17·2–18·8)	43 (39–46)	10·9 (10·0–11·7)	234 (232–235)	59·5 (59·0–60·1)
2040	72 (68–76)	13·4 (12·6–14·1)	46 (42–50)	8·5 (7·7–9·2)	259 (259–260)	48·2 (47·8–48·6)
2060	60 (56–64)	9·0 (8·3–9·6)	40 (35–44)	5·9 (5·3–6·6)	291 (289–292)	43·7 (43·5–43·8)
**Women**						
2020	92 (90–94)	18·5 (18·1–18·9)	61 (59–63)	12·2 (11·9–12·6)	252 (251–253)	50·6 (50·2–50·9)
2040	86 (83–88)	13·3 (12·9–13·7)	61 (59–63)	9·5 (9·0–9·9)	281 (280–282)	43·5 (43·2–43·9)
2060	66 (64–68)	9·0 (8·7–9·3)	49 (47–51)	6·7 (6·4–7·0)	307 (306–308)	41·9 (41·8–42·0)

Data in parentheses are 95% uncertainty intervals. Baseline hypertension scenario refers to continuing trend (on the logarithmic scale) of observed trends in hypertension prevalence 2003–17.

On the other hand, the incidence rate of disability in the population aged 65 years and older is forecasted to decrease by half between 2020 and 2060. A similar decrease is observed for dementia, with an expected fall from about 11·6 per 1000 person-years in 2020 to 6·3 in 2060.

Assuming the long-run mortality rate trend (2007–16) continues, the mortality rate among those aged 65 years and older is predicted to decrease by approximately 22% between 2020 and 2060, from about 54·5 to 42·7 per 1000 person-years. In contrast, the number of deaths is predicted to increase from about 485 000 in 2020 to 598 000 in 2060.

**Figure 1:**
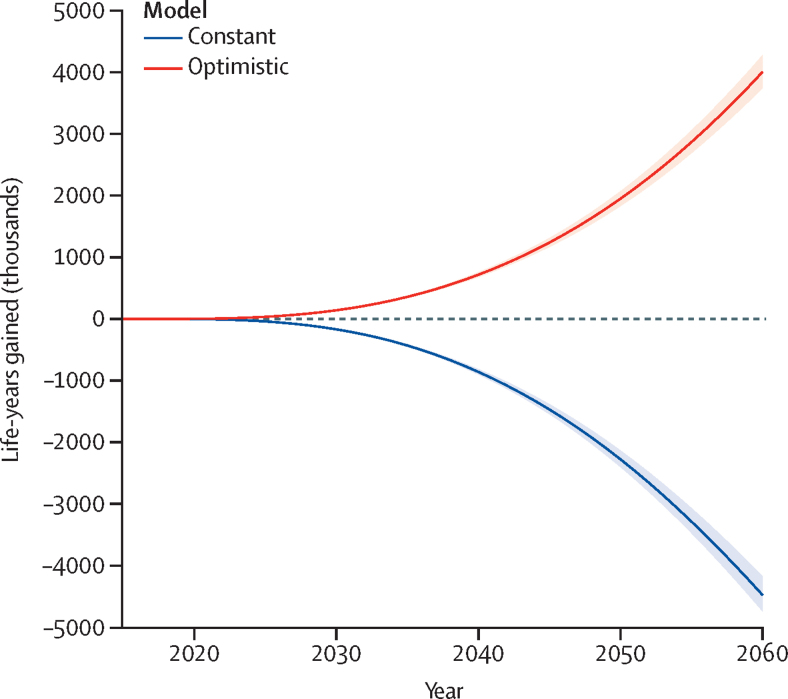
Cumulative number of life-years gained (thousands) for modelled constant and optimistic scenarios versus baseline scenario in the England and Wales population aged ≥65 years Shaded areas represent 95% uncertainty intervals.

Assuming hypertension prevalence in the last observed year (2017) remains unchanged for the period 2018–60 (constant scenario), we might expect about 81 100 additional deaths (44·7 more deaths per 100 000 population) by 2040 and approximately 177 700 additional deaths (22·9 more deaths per 100 000 population) by 2060 (table 2). This corresponds to approximately 860 000 cumulative life-years lost by 2040 and 4 479 000 by 2060 (figure 1).

**Table 2:** Number of deaths avoided (cumulative since 2015) for constant and optimistic scenarios versus baseline scenario in the England and Wales population aged ≥65 years

	**Constant scenario: hypertension prevalence remains constant**		**Optimistic scenario: 50% decrease in hypertension prevalence by 2060**	
	Deaths avoided (thousands)	Deaths avoided per 100 000 population	Deaths avoided (thousands)	Deaths avoided per 100 000 population
**All**				
2020	−1·0 (−1·1 to −1·0)	−11·5 (−11·8 to −11·3)	0·9 (0·8 to 0·9)	9·6 (9·5 to 9·7)
2040	−81·1 (−86·1 to −75·5)	−44·7 (−49·2 to −40·3)	80·7 (75·6 to 86·0)	51·5 (47·3 to 55·8)
2060	−177·7 (−193·0 to −160·2)	−22·9 (−26·6 to −19·0)	232·8 (212·7 to 253·9)	57·0 (50·4 to 63·5)
**Men**				
2020	−0·6 (−0·6 to −0·6)	−15·8 (−16·5 to −15·4)	0·3 (0·3 to 0·3)	7·6 (7·5 to 7·7)
2040	−44·7 (−48·5 to −40·6)	−51·8 (−58·1 to −45·2)	30·2 (27·4 to 32·7)	42·1 (36·9 to 46·7)
2060	−95·9 (−107·1 to −83·3)	−22·0 (−27·0 to −16·5)	80·8 (70·7 to 89·3)	36·9 (30·6 to 41·6)
**Women**				
2020	−0·4 (−0·4 to −0·4)	−8·1 (−8·2 to −7·9)	0·6 (0·6 to 0·6)	11·2 (11·1 to 11·4)
2040	−36·5 (−40·4 to −32·2)	−38·8 (−44·4 to −32·4)	50·4 (45·7 to 54·8)	59·2 (52·1 to 65·8)
2060	−82·3 (−94·1 to −68·5)	−23·8 (−29·0 to −17·6)	152·2 (132·1 to 170·0)	75·6 (62·9 to 86·4)

Data in parentheses are 95% uncertainty intervals. All values are cumulative since 2015. Negative values indicate additional burden. Baseline scenario: continuing trend (on the logarithmic scale) of observed trends in prevalence 2003–17. Constant scenario: hypertension prevalence at the last observed date (2017) would remain constant. Optimistic scenario: hypertension prevalence would be reduced by 50% by 2060.

In contrast, assuming hypertension prevalence falls by 50% between 2017 and 2060 (optimistic scenario), we might expect about 80 700 fewer deaths (51·5 fewer deaths per 100 000 population) by 2040 and approximately 232 800 fewer deaths (57·0 fewer deaths per 100 000 population) by 2060. Women would have more deaths avoided than men (table 2). The optimistic scenario leads to projected cumulative life-years gained of 717 000 and 4 006 000 by 2040 and 2060, respectively (figure 1).

For the constant scenario, we expect little change in the burdens of dementia and disability by 2060 in comparison with the baseline scenario. On the other hand, the optimistic scenario would result in a small increase in the cases of dementia but little change in the total cases of disability by 2060 compared with the baseline scenario (table 3).

**Table 3:** Number of incident cases of disability and dementia avoided (cumulative since 2015) for constant and optimistic scenarios versus baseline scenario in the England and Wales population aged ≥65 years

		**Constant scenario: hypertension prevalence remains constant**		**Optimistic scenario: 50% decrease in hypertension prevalence by 2060**	
		Cases avoided (thousands)	Cases avoided per 100 000 population	Cases avoided (thousands)	Cases avoided per 100 000 population
**Disability**					
All					
	2020	−0·2 (−0·3 to −0·2)	−2·2 (−3·3 to −1·8)	0·2 (0·1 to 0·2)	1·7 (1·4 to 2·0)
	2040	−6·6 (−10·7 to −2·7)	−1·1 (−4·2 to 1·9)	4·9 (0·5 to 8·8)	−0·5 (−3·8 to 2·7)
	2060	−13·6 (−27·7 to −0·1)	−4·8 (−9·3 to −0·6)	−1·8 (−17·7 to 13·3)	−3·0 (−7·9 to 1·7)
Men					
	2020	−0·1–0·2 to −0·1)	−2·7 (−5·4 to −2·0)	0·1 (0 to 0·1)	1·3 (1·1 to 1·6)
	2040	−2·2 (−5·6 to 1.1)	0·9 (−4·9 to 6·5)	3·1 (1·0 to 5·2)	2·5 (−1·2 to 6·4)
	2060	−1·6 (−13·6 to 10·3)	−1·0 (−9·0 to 7·3)	7·5 (−0·6 to 15·8)	6·5 (0·8 to 13·0)
Women					
	2020	−0·1 (−0·1 to −0·1)	−1·7 (−2·1 to −1·4)	0·1 (0·1 to 0·1)	2·0 (1·6 to 2·4)
	2040	−4·2 (−6·9 to −1·6)	−2·6 (−6·2 to 0·9)	1·7 (−2·0 to 5·2)	−3·1 (−8·3 to 1·9)
	2060	−11·8 (−19·8 to −3·7)	−8·3 (−12·6 to −4·1)	−9·6 (−22·5 to 2·8)	−11·9 (−19·4 to −4·6)
**Dementia**					
All					
	2020	−0·1 (−0·2 to −0·1)	−1·0 (−2·2 to −0·7)	0·1 (0 to 0·1)	0·8 (0·5 to 1·0)
	2040	3·2 (0 to 6·1)	5·3 (2·7 to 7·4)	−3·4 (−6·9 to −0·4)	−5·7 (−8·5 to −3·2)
	2060	14·6 (3·5 to 24·3)	2·8 (−0·5 to 6·0)	−24·5 (−37·5 to −12·7)	−9·0 (−13·2 to −5·1)
Men					
	2020	−0·1 (−0·2 to 0)	−1·3 (−3·9 to −0·7)	0 (0 to 0·1)	0·5 (0·3 to 0·7)
	2040	2·5 (0·1 to 5·0)	7·5 (3·7 to 11·7)	−0·4 (−1·9 to 1·0)	−2·7 (−5·4 to 0.3)
	2060	11·6 (3·4 to 20·6)	6·1 (0·3 to 12·7)	−3·7 (−9·4 to 2·9)	−1·3 (−5·6 to 3·5)
Women					
	2020	0 (−0·1 to 0)	−0·8 (−1·1 to −0·5)	0 (0 to 0·1)	0·9 (0·6 to 1·3)
	2040	0·7 (−1·2 to 2·7)	3·4 (0·8 to 6·1)	−3·0 (−6·2 to −0·2)	−8·1 (−13·0 to −4·2)
	2060	2·8 (−2·9 to 9·1)	−0·3 (−3·3 to 3·1)	−20·8 (−33·2 to −10·7)	−16·0 (−23·0 to −10·0)

Data in parentheses are 95% uncertainty intervals. All values are cumulative since 2015. Negative values indicate additional burden. Baseline scenario: continuing trend (on the logarithmic scale) of observed trends in prevalence 2003–17. Constant scenario: hypertension prevalence at the last observed date (2017) would remain constant. Optimistic scenario: hypertension prevalence would be reduced by 50% by 2060.

**Figure 2:**
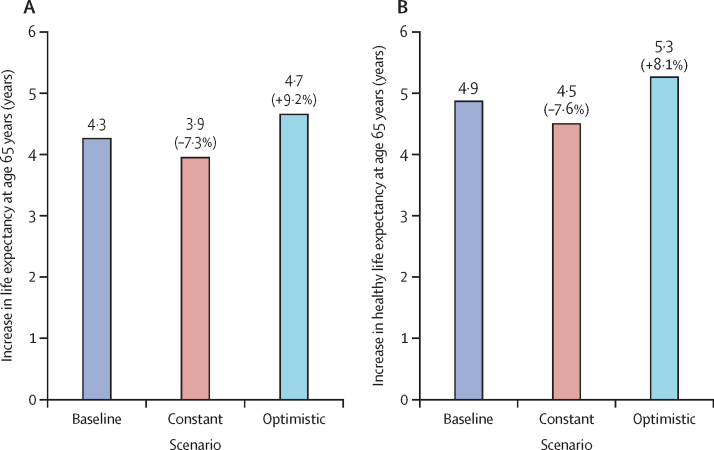
Projected increase in life expectancy (A) and healthy life expectancy (B) at age 65 years from 2020 to 2060 in England and Wales under three hypertension scenarios

For the baseline scenario, life expectancy at age 65 years is expected to increase by 4·3 years from 2020 to 2060 (16·3 years to 20·5 years). Relative to the baseline scenario, there would be a 9·2% increase in life expectancy at 65 years for the optimistic scenario, while a 7·3% decrease would be expected for the constant scenario (figure 2). Similarly, there would be an 8·1% increase in healthy life expectancy at age 65 years for the optimistic scenario and a 7·6% decrease for the constant scenario compared with the baseline scenario. Correspondingly, the percentage of life-years in the population spent with disability was 0·7 percentage points lower for the optimistic scenario compared with the baseline scenario, and 1·4 percentage points lower than the constant scenario. The percentage of life-years in the population spent with dementia was 0·02 percentage points lower for the optimistic scenario compared with the baseline scenario, and 0·08 percentage points lower than the constant scenario (appendix p 28).

For the sensitivity analyses, seven assumptions were made about the future mortality trend, most pessimistically from no improvement in mortality rate to, optimistically, a doubling in the declining mortality rate trend, compared with that in 2010–16. These analyses showed similar results to the main analyses, with more pessimistic mortality scenarios (slower mortality rate reduction) having slightly more life-years gained in the optimistic hypertension prevalence scenario and more life-years lost in the constant scenario compared with the baseline scenario (appendix p 29). Similar impact was observed in the life-years spent with disability and dementia. More pessimistic mortality scenarios result in fewer life-years spent with disability and dementia in all the hypertension prevalence scenarios (appendix pp 30–31). However, more pessimistic mortality scenarios lead to slightly more life-years gained free of disability and dementia in the optimistic scenario and more life-years lost in the constant scenario compared with the baseline scenario (appendix p 32).

## Discussion

Using a well validated Markov model that integrates competing influence of mortality and morbidity trends, we found that the major impact of alternative trends of future hypertension prevalence appears to be on future life expectancy. The effect on future dementia and disability burden appears to be modest or trivial. Importantly, a 50% reduction of hypertension prevalence by 2060 from its 2017 level would lead to an additional 8% increase (5·3 years *vs* 4·9 years) in healthy life expectancy at age 65 years and projected cumulative life-years gained of 4 006 000 by 2060.

An early review study^25^ suggested approximately 5% (1·7 million) of worldwide cases of Alzheimer's disease were attributable to midlife hypertension. Using a basic population-attributable-risk method, they estimated that 10% and 25% reduced prevalence of midlife hypertension could result in 40 000 and 100 000 fewer Alzheimer's disease cases worldwide, respectively. Their estimates emphasise how hypertension prevention strategies might reduce the number of dementia cases. However, to predict reliably the effect of interventions to reduce hypertension on future dementia prevalence, population ageing, mortality improvement, and the correlation between risk factors need to be taken into account. A more recent modelling study considered the inter-relation of dementia risk factors,^26^ again predicting major potential benefit, such that a modestly declining future trend in midlife hypertension and other risk factors would reduce the worldwide prevalence of Alzheimer's disease by between 8% and 15% (8·8 million and 16·2 million cases) and reduce the prevalence in the UK by between 9% and 16% (170 000 and 314 000 cases) in 2050. However, the benefit might be overestimated because the study did not consider reduction of mortality by hypertension prevention strategies, which are likely to increase the number of older people susceptible to dementia and time spent living with dementia.

In contrast, the present study predicts an inverse relation between hypertension prevalence and number of dementia cases in the UK—ie, declining future midlife hypertension associated with an increase in the number of people living with dementia. Our estimate is based on a multistate model that synthesises important competing epidemiological trends, particularly in CVD incidence, and CVD and non-CVD mortality, together with the increasing size of the older population.^1^, ^17^, ^18^, ^27^ This method showed that the ageing demographic trend will be the major determinant of dementia burden in the coming decades.^1^ In respect of the apparent paradox of declining hypertension and increasing dementia occurrence, our modelling study reflects the evolving impact of the intervention scenarios on incidence of CVD and mortality, and suggests that the salutary effect of lower population blood pressure distribution on incidence of dementia might not offset expansion of the susceptible population due to reduced mortality.

Another important health state in older people is disability. Using a macro-simulation model with transition probabilities estimated from the first Medical Research Council Cognitive Function and Ageing Study (MRC CFAS), one study projected over 700 000 more cases of disability over 20 years in England and Wales^28^ and another study projected 550 000 more cases of functional disability over 30 years in England.^29^ Both studies are based on the MRC CFAS set up 30 years ago, and model input parameters such as dementia incidence might not reflect the current trend. Our recent study based on a multistate model with estimates from ELSA predicted 560 000 additional disability cases in 2025 compared with 2015.^17^ However, little is known about how hypertension intervention would affect the predicted number of disability cases, and the present study found that a reduction in prevalence of hypertension by half from its 2017 level to 2060 would not greatly decrease the overall disability burden. The potential explanation is that the beneficial effect of hypertension intervention on disability is offset by increased burden of dementia and its related disability due to reduced mortality.

Robust estimates for the effect of hypertension prevention on future burden of dementia and disability need to consider the complex population dynamics of lifespan and interactions between hypertension-associated diseases such as dementia, disability, and CVD. One key strength of our study is that the structure of the IMPACT-BAM model allows us to take into account the complex interactions between those hypertension-associated diseases as well as calendar trends in the incidences of those diseases. Changes in mortality rates are also considered in the model. If the current observed stalling progress in slowdown of mortality rates continues, hypertension prevention will lead to substantial gains in total life-years and life-years free of dementia and disability. Another strength is that hypertension prevalence and relative risks for the effect of hypertension on various health outcomes are based on the best available evidence from recent systematic reviews and meta-analyses.

Our study has limitations. We used the PARF approach to translate trends in hypertension prevalence to changes in the risk of dementia and disability. The Levin's formula for calculating PARF and related PIF in our study is biased if adjusted RR is used in the formula. However, this does not undermine the usefulness of the method because the main objective of our study was to estimate the relative impact of scenarios involving different hypertension prevention strategies, rather than the precise estimation of the absolute impact. The PARF approach assumes causality between risk factors and disease. Meta-analysis of randomised clinical trials shows that blood pressure lowering with antihypertensive agents is associated with a lower risk of incident dementia or cognitive impairment.^30^ Also, the PARF approach relies on other assumptions including, but not limited to, that other risk factors are unaffected and that there are no unmeasured confounders.^24^ Violation of these assumptions will affect our projection in each hypertension prevalence scenario, but is less likely to change the conclusion from the comparison of different scenarios. Disabilities caused by conditions other than CVD and dementia are aggregated into a single model state. Transition probabilities of moving from this state to other states are non-specific and represented by an average probability. Nevertheless, the estimated combined probabilities should be generalisable since the sample from ELSA is representative for the population of England and Wales. Lastly, our approach has not accounted for the effect of changes in the distribution of risk factors other than hypertension, and therefore our results might be conservative.

We extrapolate the observed incidence trend of dementia, disability, and CVD to 2060 to correspond to the biology and time course of the effect of midlife hypertension on dementia risk.^10^ It is clear that the further ahead the modelled prediction is made, the more uncertainty there is about the future epidemiological trends. We tested the sensitivity of our model predictions to key assumptions such as the future mortality rate trend (appendix pp 29–32). We also addressed uncertainty in the real data as a source of uncertainty in the projected dementia burden and other predictions using Monte Carlo simulation.

Our findings have several public health implications. Through the next 40 years, we might expect a shift in the burden of hypertension consequences, from CVD towards dementia. Our study suggests that reduction in the prevalence of hypertension is important in the context of the goal of healthy ageing, not only reducing the future burden of CVD, but also compressing the future burden of disability and shifting the burden of dementia towards the end stage of life. Furthermore, as CVD mortality continues to decline, there could be a further shift in the burden of hypertension to non-cardiovascular complications. Evidence suggests that upstream, structural interventions are powerful prevention strategies. Several UK policies now aim to prevent the rise in hypertension prevalence, including a high blood pressure action plan^31^ and Action on Salt. In terms of individual-level intervention, the UK National Health Service currently offers regular blood pressure checks and gives advice to promote a healthier lifestyle for adults in England aged 40–74 years. Education interventions need to be implemented to raise awareness that a healthy lifestyle can prevent dementia, as nearly half of adults older than 50 years are unaware of this.^32^ On the other hand, the important negative finding that better hypertension intervention is not expected to significantly affect future burden of dementia and disability indicates that other preventive interventions targeting risk factors such as obesity, alongside hypertension prevention strategies, might better reduce the burdens of dementia and age-related disability.

In conclusion, our study showed that reduction in hypertension prevalence would result in additional gain in future life expectancy at age 65 years, but have little effect on disability burden and paradoxically could increase the dementia burden. Our study suggests that hypertension prevention strategies can improve healthy ageing and emphasises the urgent need for policy development that includes multidomain interventions to mitigate the burden of dementia and disability.

## Data sharing

The data involved in this analysis are available through the UK Data Service (through which data from ELSA [elsa-project.ac.uk] and hypertension prevalence data from the Health Survey for England can be accessed). A technical appendix with details on the formula and calculations for the IMPACT-BAM model is available from the corresponding author.

## Declaration of interests

SA-A acts as an unpaid advisor for Medsien. All other authors declare no competing interests.
