# Supplementary material for: Impact of hypertension prevalence trend on mortality and burdens of dementia and disability in England and Wales to 2060: a simulation modelling study
Source: Lancet Healthy Longev. 2023 Sep;4(9):e470–7. doi: 10.1016/S2666-7568(23)00129-0 (PMC11108803; doi:10.1016/S2666-7568(23)00129-0)
Supplement: Supplementary appendix [file mmc1.pdf]

THE LANCET
Healthy Longevity

Supplementary appendix

This appendix formed part of the original submission and has been peer reviewed. We post it as supplied by the authors.

Supplement to: Chen Y, Araghi M, Bandosz P, et al. Impact of hypertension prevalence trend on mortality and burdens of dementia and disability in England and Wales to 2060: a simulation modelling study. *Lancet Healthy Longev* 2023; published online Aug 10. https://doi.org/10.1016/S2666-7568(23)00129-0.

Supplementary Materials

Impact of hypertension prevalence trend on mortality and burdens of dementia and disability in England and Wales to 2060: a simulation modelling study

Contents:

Overview of IMPACT-BAM model and Matrix calculation 2
Future trends in hypertension prevalence 20
Mortality projections 24
Literature review 26
Effect on life expectancy and compression of morbidity 28
Sensitivity analysis for future mortality trends 29
Reference 33

Overview of IMPACT-BAM model and Matrix calculation

(Note this part mainly reproduced from Guzman-Castillo et al., 2017 supplementary material[1])

The IMPACT Better Ageing Model (IMPACT-BAM) is a discrete-time Markov model which follows the progression of a healthy population (aged 35+ years old) from England and Wales into ten different health states characterised by the presence or absence of cardiovascular disease (CVD), cognitive impairment and functional impairment from 2006 to 2060. The model structure is presented in **Figure S1**, the health states are described in **Table S1** and transition probabilities, $p_{i,j}$, in **Table S2**.

Prior to simulation, we populated each state in the model based on ONS population estimates in 2006 (start year) and prevalence of the above conditions from ELSA, except for the new cohort of 35-year-olds that enters the system through the disease-free state. The simulation allows individuals to move to other states in the model. The arrows in **Figure S1** indicate the possible movements of people between these ten states, which are governed by one-year probabilities of transition.

For example, a healthy 55-year man starts the simulation in state 1 (Disease-free state) in 2006. He moves to state 2 (CVD) in 2007 after having a stroke. In 2008 he could either die from complications of the stroke (he moves to state 9), any other causes (he moves to state 10) or he could develop cognitive impairment (moving to state 3) or disability (moving to state 5). As above, movements to any state are driven by transition probabilities. Detailed information on the estimation of transition probabilities is provided in the following **Input and Calculations** section.

Then, to calculate the number of people with disability in year t, we count the number of people in the states that represent disability (states 5, 6, 7 and 8) at year t. To calculate the number of individuals with dementia, we count the number of people in state 6 and 7 at year t, and do so similarly for other conditions. These numbers are then used to calculate the prevalence of any disease in the model. Detailed information on output calculation can be found in **Table S3**.

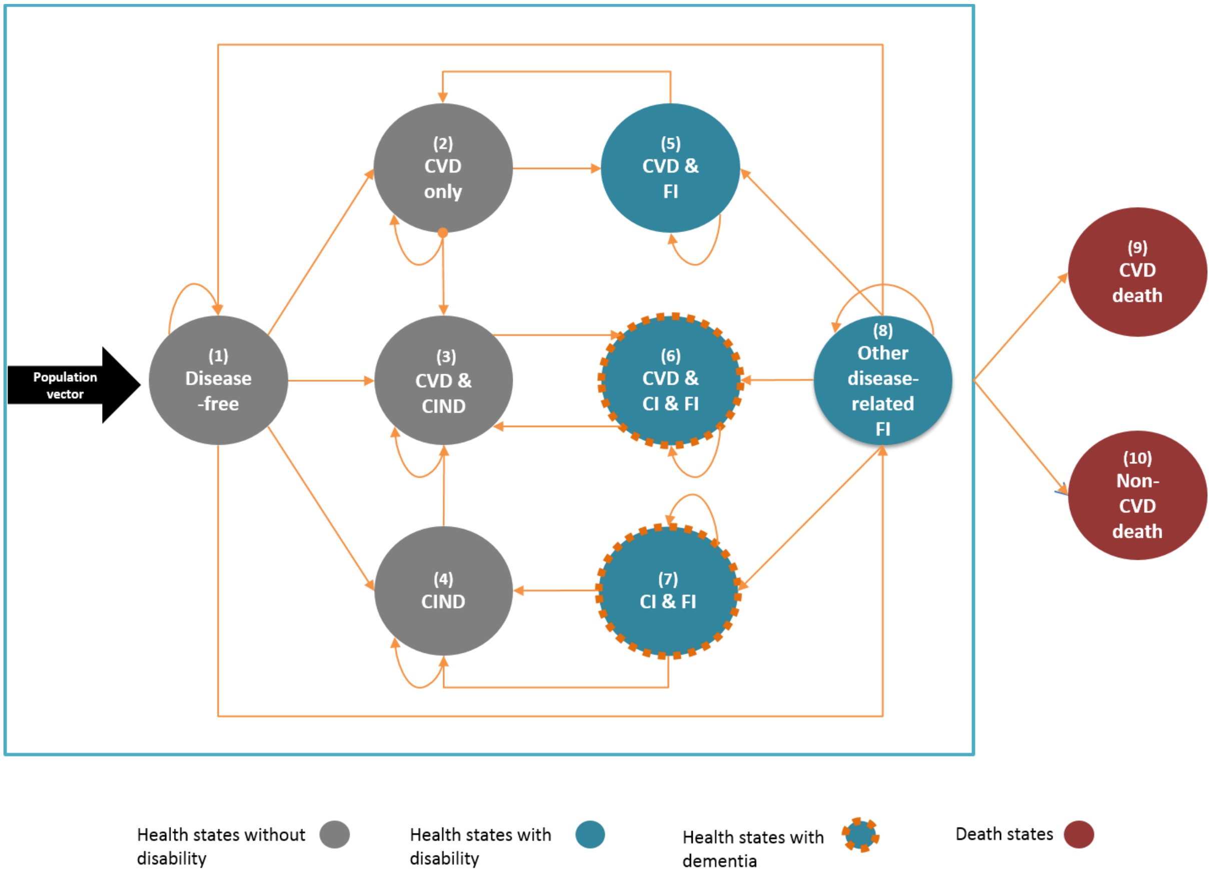

Figure S1 IMPACT-BAM model structure. Transitions to death states 9 and 10 are possible from any state.

Footnote

CVD: cardiovascular disease; CI: cognitive impairment; CIND: cognitive impairment no dementia; FI: functional impairment

Table S1 Description of the health states

Health state	Name	Description
1	Disease-free population	People free of cardiovascular disease (CVD), cognitive impairment (CI) or functional impairment (FI)
2	CVD only	Cardiovascular disease
3	CVD and CIND	Cardiovascular disease and cognitive impairment no dementia
4	CIND	Cognitive impairment no dementia
5	CVD and FI	Cardiovascular disease and functional impairment
6	CVD, CIND and FI	Cardiovascular disease and dementia (cognitive + functional impairment)
7	DEMENTIA	cognitive + functional impairment
8	Other disease-related FI	Functional impairment no related to CVD or/and Dementia
9	CVD death	Death from CVD causes
10	Non-CVD death	Death from a different cause than CVD

Table S2 Description of transition probabilities. Each transition probability is stratified by sex and age

1-year transition probability	From	To
$p_{1,1}$	Disease-free population	Disease-free population
$p_{1,2}$	Disease-free population	CVD only
$p_{1,3}$	Disease-free population	CVD and CIND
$p_{1,4}$	Disease-free population	CIND
$p_{1,8}$	Disease-free population	Non-CVD/Non-dementia FI
$p_{1,9}$	Disease-free population	CVD death
$p_{1,10}$	Disease-free population	Non-CVD death
$p_{2,2}$	CVD only	CVD only
$p_{2,3}$	CVD only	CVD and CIND
$p_{2,5}$	CVD only	CVD and FI
$p_{2,9}$	CVD only	CVD death
$p_{2,10}$	CVD only	Non-CVD death
$p_{3,3}$	CVD and CIND	CVD and CIND
$p_{3,6}$	CVD and CIND	CVD and Dementia
$p_{3,9}$	CVD and CIND	CVD death
$p_{3,10}$	CVD and CIND	Non-CVD death
$p_{4,4}$	CIND	CIND
$p_{4,7}$	CIND	Dementia
$p_{4,9}$	CIND	CVD death
$p_{4,10}$	CIND	Non-CVD death
$p_{5,5}$	CVD and FI	CVD and FI
$p_{5,2}$	CVD and FI	CVD only
$p_{5,9}$	CVD and FI	CVD death
$p_{5,10}$	CVD and FI	Non-CVD death
$p_{6,6}$	CVD and dementia	CVD and dementia
$p_{6,3}$	CVD and dementia	CVD and CIND
$p_{6,9}$	CVD and dementia	CVD death
$p_{6,10}$	CVD and dementia	Non-CVD death
$p_{7,7}$	Dementia	Dementia

$p_{7,4}$	Dementia	CIND
$p_{7,9}$	Dementia	CVD death
$p_{7,10}$	Dementia	Non-CVD death
$p_{8,1}$	Other disease-related FI	Disease-Free population
$p_{8,5}$	Other disease-related FI	CVD and FI
$p_{8,6}$	Other disease-related FI	CVD and Dementia
$p_{8,7}$	Other disease-related FI	Dementia
$p_{8,8}$	Other disease-related FI	Non-CVD/Non-dementia FI
$p_{8,9}$	Other disease-related FI	CVD death
$p_{8,10}$	Other disease-related FI	Non-CVD death

1 Input and Calculations

1.1 Incidence of CVD ($P_{1,2}$, $P_{4,3}$, $P_{8,5}$)

Denote $P(CVD)$ to be the incidence of CVD. To calculate CVD incidence, $P(CVD)$, we obtained 2-year incidence rates from the English Longitudinal Study of Ageing (ELSA) and fitted a logistic regression model of the form:

$$logit\ incidence = \beta_0 + \beta_{age} age_{35} + \beta_{sex} sex + \beta(sex * age_{35}) + \beta_{state} state$$

Where age_{35} is individual age centred at 35 and *state* are those states (states 1, 4 and 8) from where transitions to CVD states (states 2, 3 and 5) are allowed.

From the logistic regression estimates, 2-year transition probabilities were computed which were later transformed into gender specific 1-year transition probabilities for single years of age.

In our model, we defined states 2 and 4 (CVD-only and CIND-only) as mutually exclusive (i.e., a patient who is in the CVD-only state does not have CIND at the same time and vice versa). Therefore, to calculate the transition probability $p_{1,2}$, we subtract the proportion of patients who have both CVD and CIND, $p_{1,3}$

$$p_{1,2} = P(CVD) - p_{1,3}$$

1.2 Incidence of CIND ($P_{1,4}$, $P_{2,3}$, $P_{8,7}$)

Denote $P(CIND)$ to be the incidence of "cognitive impairment no dementia". To calculate CIND incidence, $P(CIND)$, 2-year incidence rates from ELSA were modelled as follows:

We fitted a logistic regression model of the form:

$$logit\ incidence = \beta_0 + \beta_{age} age_{50} + \beta_{sex} sex + \beta(sex * age_{50}) + \beta\left(sex * age_{50}^2\right) + \beta_{state} state$$

Where age_{50} is individual age centred at 50 and *state* are those states (states 1, 2 and 8) from where transitions to CI states (states 4, 3 and 7) are allowed.

This allowed us to compute 2-year transition probabilities that were later transformed into gender specific 1-year transition probabilities for single years of age. The incidence rates from ELSA are likely to be underestimated due to higher drop out of those who do develop cognitive impairment.

In our model, we defined states 2 and 4 (CVD-only and CIND-only) as mutually exclusive (i.e., a patient who is in the CVD-only state does not have CIND at the same time and vice versa). Therefore, to calculate the transition probability $p_{1,4}$, we subtract the proportion of patients who have both CVD and CIND, $p_{1,3}$

$$p_{1,4} = P(CIND) - p_{1,3}.$$

1.3 Incidence of CVD and CIND ($P_{1,3}$, $P_{8,6}$)

We assume that CVD and CIND are independent events. Therefore, ($p_{1,3} = P(CVD \cap CIND)$) from a healthy state, $p_{1,3} = P(CVD) \times P(CIND)$ from above formula.

Similarly for $p_{8,6}$

1.4 Incidence of functional impairment states ($P_{1,8}$, $P_{2,5}$, $P_{3,6}$, $P_{4,7}$)

We obtained the 2-year incidence rates for functional impairment ELSA and fitted logistic regression models of the form:

$$logit\ incidence\ FI = \beta_0 + \beta_{age} age_{35} + \beta_{sex} sex + +\beta_{state} state + \beta(sex * age_{35}) + \beta(state * age_{35})$$

Where $age_{35}$is individual age centred at 35 and *state* are those states (states 1, 2, 3 and 4) from where transitions to FI states (states 8, 5, 6 and 7) are allowed.

This allowed 2-year transition probabilities to be computed which were later transformed into gender specific1-year transition probabilities for single years of age. These transition probabilities do not have a calendar effect.

1.5 Recovery from functional impairment states ($P_{8,1}$, $P_{5,2}$, $P_{6,3}$ and $P_{7,4}$)

We obtained the 2-year incidence rates for functional impairment ELSA and fitted logistic regression models of the form:

$$logit\ incidence\ FI = \beta_0 + \beta_{age} age_{35} + \beta_{sex} sex + +\beta_{state} state + \beta(sex * age_{35}) + \beta(state * age_{35})$$

Where $age_{35}$is individual age centred at 35 and *state* are those FI states (states 8, 5, 6 and 7) from where transitions to states without FI (states 1, 2, 3 and 4) are allowed.

This allowed 2-year transition probabilities to be computed which were later transformed into gender specific1-year transition probabilities for single years of age. These transition probabilities do not have a calendar effect.

1.6 Transition probabilities from state i to the death states ($P_{i,9}$, $P_{i,10}$)

The computation of the transition probabilities $p_{i,9}$ involved three steps:

For the first step, CVD mortality probabilities of CVD up 2025 in 5-year age bands were calculated using the Bayesian Age Period Cohort (BAPC) model,[2] with ONS mortality and population estimates from 1982-2012 for England and Wales as inputs.

The curve fitting tool in MATLAB was then used to obtain CVD mortality probabilities for single years of age, starting at 35 years old. The probabilities are estimated using piecewise cubic Hermit interpolation to estimate values that lie between known data points, with the monotonicity and the shape of the data preserved. We denote these probabilities of death by $m\_cvd_{a,t}$, where *a* is the age of individual and *t* the calendar year.

For the second step, we calculated mortality rates from ELSA for the age groups 50-59, 60-69, 70-79 and 80-89 and fitted two logistic regression models of the form:

$$logit\ cvd\_death = \beta_0 + \beta_{age} age_{35} + \beta_{male} male + \beta(male * age_{35})$$

$$logit\ cvd\_death = \beta_0 + \beta_{age} age_{35} + \beta_{male} male + \beta(male * age_{35}) + \boldsymbol{\beta_s} \mathbf{state}$$

Where $age_{35}$is individual age centred at 35, $\boldsymbol{\beta_s}$ is a vector containing the β coefficients for all the states.

The first equation allowed us to compute gender specific baseline transition probabilities for single years of age. We defined these as $\tilde{p}_{0,9,a}$

The second equation allowed us to compute gender and state-specific transition probabilities for single years of age. We defined these as $\tilde{p}_{i,9,a}$

To estimate how different the state-specific transition probabilities are from the baseline transition probabilities we calculated $cvd_{a,i} = \frac{\tilde{p}_{i,9,a}}{\tilde{p}_{0,9,a}}$.

The probabilities of death, $m\_cvd_{a,t}$, are the probabilities of dying (from CVD) regardless of the state an individual is coming from, similar to the baseline transition probabilities $\tilde{p}_{0,9,a}$ from the ELSA study. The $m\_cvd_{a,t}$ are calculated using the entire England and Wales population and allow for cohort and calendar effects and are preferred over the $\tilde{p}_{0,9,a}$.

To allow for each subject's initial state, the $m\_cvd_{a,t}$ were multiplied by the factor $cvd_{a,i}$ to obtain the age, gender and state-specific transition probabilities $p_{i,9,a}$.

Transition probabilities $p_{i,10,a}$ were calculated in the same manner.

1.7 Calendar effect for CVD and CIND incidence

Let $\Delta_{a,t+1}= \frac{m\_cvd_{a,t+1}}{m\_cvd_{a,t}}$ where $m\_cvd_{a,t}$ is the age-specific probability of death from CVD causes in year *t*. Therefore, $\Delta_{a,t+1}$ is an age-specific adjustment factor describing how different the probability of CVD death in year *t+1* is from the probability of CVD death in the previous year *t*.

We assume that annual changes in CVD incidence mirror the annual changes in CVD mortality as observed in ELSA. In other words, we assume the annual percentage change in CVD incidence equals to the annual percentage change in CVD mortality. Therefore, to obtain the incidence of CVD allowing for a calendar effect, we multiplied $P(CVD)_{a,t+1}$ by $\left(\Delta_{a,t+1}\right)$.

Likewise, we assume that these annual changes in CVD incidence would also affect $p_{8,5}$, thus the same calendar was applied.

However, the incidence of CIND, $P(CIND)_{a,t+1}$, is assumed to decrease by 2.7% per calendar year, i.e., $P(CIND)_{a,t+1}$ = 0.973 * $P(CIND)_{a,t}$.

The above annual decline for CIND was estimated with data collected over 6 waves of ELSA (2002-2013) and using an elaborate model that takes into account losses to follow-up and mortality. The results of these analyses suggested that the calendar trend per year is -2.7 (*95% confidence interval* -2.9, -2.4) %.[3]

Likewise, we assume an annual 2.7% decrease for $p_{8,6}$ and $p_{8,7}$.The calculations of $p_{1,2}, p_{1,3}$ and $p_{1,4}$, proceed as previously described.

1.8 Recurrent state transition probabilities

The recurrent state transition probabilities such as $p_{1,1}$, $p_{2,2}$, $p_{3,3}$ etc. were calculated using the following formula:

$p_{i,i} = 1 - \sum_{j=1}^{J} p_{i,j}$, where *J* is a vector containing the states (other than *i* itself) to where a transition from state *i* is possible.

1.9 Prevalence of initial states

We obtained the 2-year prevalence rates for states 2, 3, 4, 5, 6, 7 and 8 from ELSA for 5-year age groups. Due to the small number, it was assumed that those aged <50 have a prevalence probability of cognitive impairment equal to zero. This was done by dividing the number of people in each state by the total number of individuals in that age-sex strata in the pooled ELSA data and attributed to 2006 which is the mid-point of the ELSA data collection period (2002-2013).

We then used the curve fitting tool in MATLAB to obtain data for single year of age starting at 35 years old.

ELSA contains information on 48 individuals aged 34 to 36. 47 (98%) of these individuals were free of CVD, cognitive impairment, dementia and disability. Therefore, we assumed that the new cohort of 35s entering the model at each year is free of disease.

2. Matrix calculation

The following table contains the steps to calculate the Markov model.

Table S3 Matrix notation for programming purposes mainly (Example for men)

Matrix formulation (Example for men)	Description
$\mathbf{p\_m_a} = [\mathrm{p\_m_{a,1}}, \mathrm{p\_m_{a,2}}, \dots, \mathrm{p\_m_{a,10}}]$	Column vector containing prevalence rates for all states for men aged *a*
$M_{a,0}$	Scalar containing initial population men aged *a*
$\mathrm{Tm_{a,t}} = \begin{bmatrix} p_{1,1,a,t} & \cdots & p_{1,10,a,t} \\ \vdots & \ddots & \vdots \\ p_{10,1,a,t} & \cdots & p_{10,10,a,t} \end{bmatrix}$	Matrix for men aged *a*, containing the transition probabilities
$\mathbf{m_{a,t}} = [\mathrm{m_{a,t,1}}, \mathrm{m_{a,t,2}}, \dots, \mathrm{m_{a,t,10}}]$	Column vector containing the number of men aged *a* in each state at time *t*
For $t = 0$ $\mathbf{m_{a,0}} = M_{a,0} \cdot \mathbf{p\_m_a}$	
For $t = n$ $\mathbf{m_{a,t}} = \mathbf{m_{a-1,t-1}} \cdot [\mathrm{T_{a-1}}]^{\mathrm{T}}$ $\mathbf{m_{a,t}} = \begin{bmatrix} m_{a-1,t-1}p_{1,1,a-1,t-1} + m_{a-1-1,t-1}p_{2,1,a-1,t-1} + \cdots + m_{a-1,t-1}p_{10,1,a-1,t-1}, \\ m_{a-1,t-1}p_{1,2,a-1,t-1} + m_{a-1,t-1}p_{2,2,a-1,t-1} + \cdots + m_{a-1,t-1}p_{10,2,a-1,t-1}, \\ \dots, \\ m_{a-1,t-1}p_{1,10,a-1,t-1} + m_{a-1,t-1}p_{2,10,a-1,t-1} + \cdots + m_{a-1,t-1}p_{10,10,a-1,t-1} \end{bmatrix}$	

Table S4 Summary of assumptions underlying the IMPACT-BAM model

	Assumption	Explanation/Evidence
IMPACT-BAM models health transitions of the population of England and Wales aged 35 and over through to death. The input data required by the IMPACT-BAM probabilistic Markov model are the starting population numbers in each age and sex strata, starting prevalence values, and transition probabilities by age, sex, and calendar year.		
**Population Numbers by Age and Sex**		
	Estimates for population numbers by sex and 5-year age-groups are obtained from the UK Office for National Statistics (ONS). At each one calendar year iteration of the model, men and women reaching age 35 are entered. The predictions for number of people aged 35 are obtained from the ONS.<br>Assumption: The model does not account for immigration to or emigration from England and Wales.	The UK Office for National Statistics provides official estimates for population demographics.
**Starting Prevalence values**		
	Initial prevalence of all health states in the model by age and sex are obtained from the English Longitudinal Study of Ageing (ELSA).<br>Assumption: ELSA is a representative sample of the population of England and Wales.	Accuracy of prevalence values depends on how well ELSA participants are a representative sample of the population of England and Wales. ELSA study participants aged 50 and over were selected at random. The core participants' cohabiting partners, including adults aged below 50, were also enrolled in the study. The overall response rate was 67%. To ensure study participants form a representative sample, survey weights are applied. To maintain representativeness at every phase of data collection, refreshment samples are recruited to the study periodically. Comparisons of the sociodemographic characteristics of participants against results from the national census indicated that the ELSA sample was broadly representative of the English population.
	To improve statistical power, six waves of ELSA data were pooled together and prevalence estimates (of CVD, cognitive impairment and functional impairment that define the health states) attributed to the mid-point of the data collection time-frame (2006). The year 2006 is therefore the starting point of the IMPACT-BAM Markov model.	The prevalence values obtained from the pooled data matched the prevalence values obtained at wave three which was the mid-point of the data collection time-frame.<br>Estimates for prevalence of cardiovascular disease are displayed as an example:

<table>
<tr>
<td></td>
<td>Assumption: Prevalence estimates from 6 waves of data pooled together correspond to prevalence estimates at mid-point of data collection – year 2006.</td>
<td>
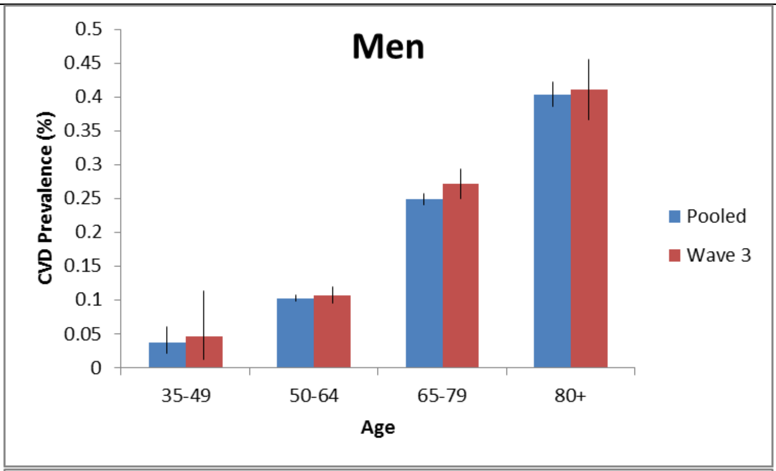

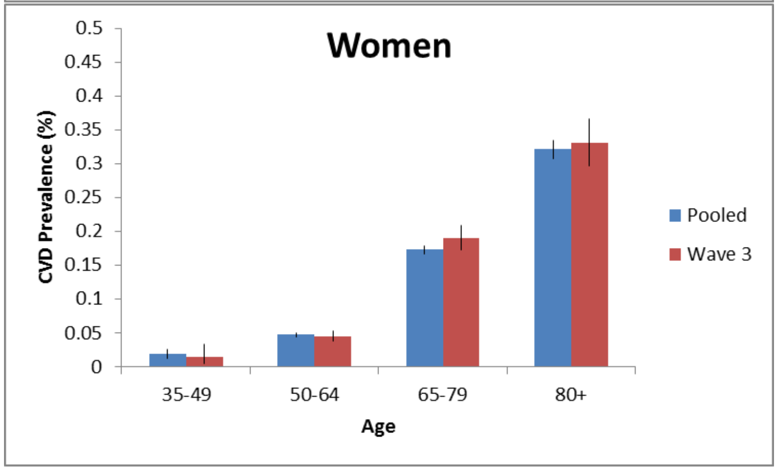

</td>
</tr>
<tr>
<td></td>
<td>The Prevalence of each health state at each calendar year from the starting point (2006) onwards equals number of persons who were in that health state in the previous year, plus new incident cases, minus those who made the transition to another health state or died from any cause. The number of new incident cases and numbers of death are determined</td>
<td>Epidemiologic concept applied to Markov models.</td>
</tr>
</table>

	by transition probabilities to and from that condition.	
**Transition Probabilities**		
	Transition probabilities were obtained as a function of age and sex from incident cases between wave n and n+1 in ELSA. As with estimates of prevalence values, the transition probabilities obtained from pooling ELSA epochs are attributed to the mid-point of the data collection period.<br>Assumption: ELSA is a representative sample of the population of England and Wales, thus transition probabilities, (equivalent to incidence by age/sex/calendar year) for cardiovascular disease, dementia, functional impairment, and mortality rates in ELSA are similar to those for England and Wales.	Incidence of cardiovascular disease and dementia by age and sex were consistent with age, and sex specific incidence values obtained from independent external sources for the corresponding calendar time. As an example, we show data related to dementia incidence below:<br>Estimates of incidence of dementia in the UK were available from the cognitive function and ageing study-II (CFAS II) between years 2008 and 2011. Incidence of dementia in the corresponding time-frame in ELSA (2008-2013) is comparable with CFAS-II estimates as displayed in the figures below.

<table>
<tr><td></td><td></td><td>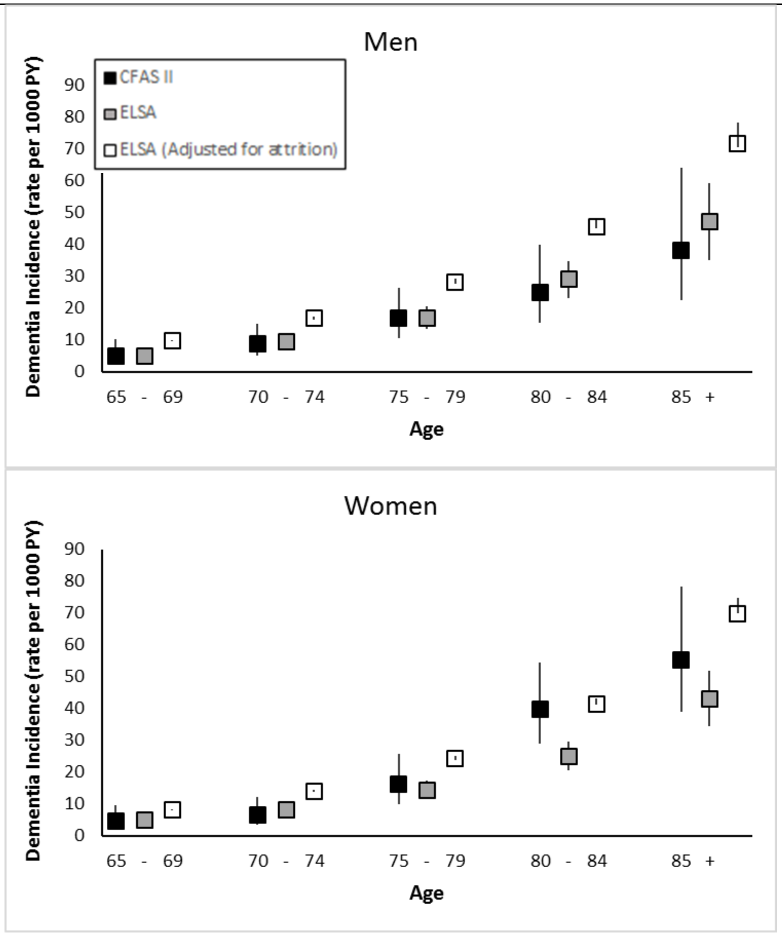

Numbers of deaths predicted from the IMPACT-BAM model matched with observed and predicted mortality rates from the ONS (Figure S3).</td></tr>
<tr><td></td><td>Assumption: Transition probabilities are a weighted average across the spectrum of the severity of each condition thus varying severities among people in each health state is accounted for.</td><td>The probability of death or development of functional impairment among those with cardiovascular disease or cognitive impairment is dependent on the severity of cardiovascular disease or cognitive impairment. Under the assumption that ELSA participants are a representative sample of the population (see above), it is reasonable to assume the spectrum of the severity of conditions (cardiovascular disease or cognitive</td></tr>
</table>

		impairment) observed in ELSA is proportionate to the spectrum of the severity of the conditions at the population level. As such, it is reasonable to assume transition probabilities obtained from ELSA are a weighted average of the transition probabilities across the spectrum of the severity of the conditions.<br>The weighted average transition probability multiplied by the total number of people in a health state is mathematically equivalent to the sum of the product of severity specific transition probabilities and severity specific numbers of people in that health state.<br>Similarly, survival of persons with each condition is assumed to be equivalent to the weighted average of survival of persons with different levels of severity.
	Assumption: The effect of comorbidities (such as diabetes) is accounted for in the model.	Since ELSA participants are assumed to be a representative sample of the population of England after weighting (see above), estimates for risks of dementia, cardiovascular disease, functional impairment and death obtained from ELSA reasonably represent a weighted average of risk levels across the spectrum of the severity of these conditions and comorbidities.
**Calendar trends:**<br>Transition probabilities (mortality rates and incidence of cardiovascular disease and dementia) change over time.		
	Assumption: Observed calendar trend in mortality rates from 2007 to 2016 is likely to continue to the future.	We considered the observed stalling progress in life expectancy in UK after 2010. We used 7-year (2010-2016) instead of 10 year (2007-2016) observed mortality data to predict future mortality rates. Furthermore, six additional mortality trend scenarios (most pessimistically from no improvement in mortality rate to, optimistically, a doubling in the declining mortality rate trend compared to that in 2010-2016) were modelled to estimate future dementia and disability trends.
	Assumption: Trends in incidence of cardiovascular disease over time are parallel to cardiovascular mortality.	Age and sex standardised cardiovascular incidence and mortality rates declined in parallel in ELSA, as below:

		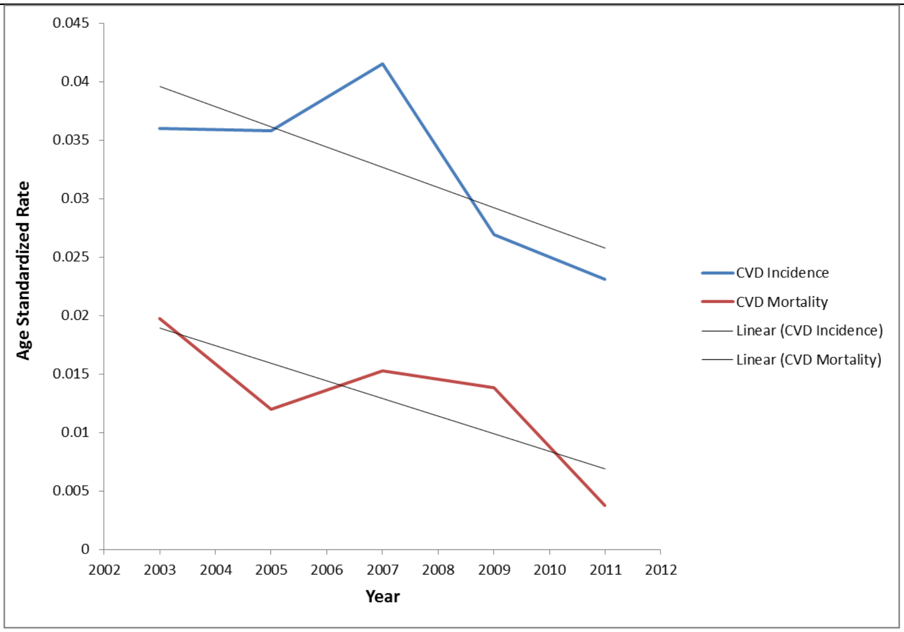<br>In response to uncertainty of this assumption, we conducted a sensitivity analysis assuming incidence of cardiovascular disease does not decline any further. Sensitivity analysis assuming CVD incidence does not decline had only a small impact on the predicted numbers of people living with dementia in the future (Figure S4).
	Assumption: Dementia incidence declines over time.	A decline in dementia incidence has been reported in several studies. The magnitude of the calendar trend is less certain. As the more likely scenario we applied calendar trends corrected for deaths and loss to follow up of study participants obtained from robust statistical modelling of ELSA data.[3] We conducted sensitivity analyses with calendar trends estimated from other studies (including CFAS I and II, and the Rotterdam study), and a worst-case-scenario in which dementia incidence does not decline any further. The figure below shows the number of dementia cases predicted in these sensitivity analyses.

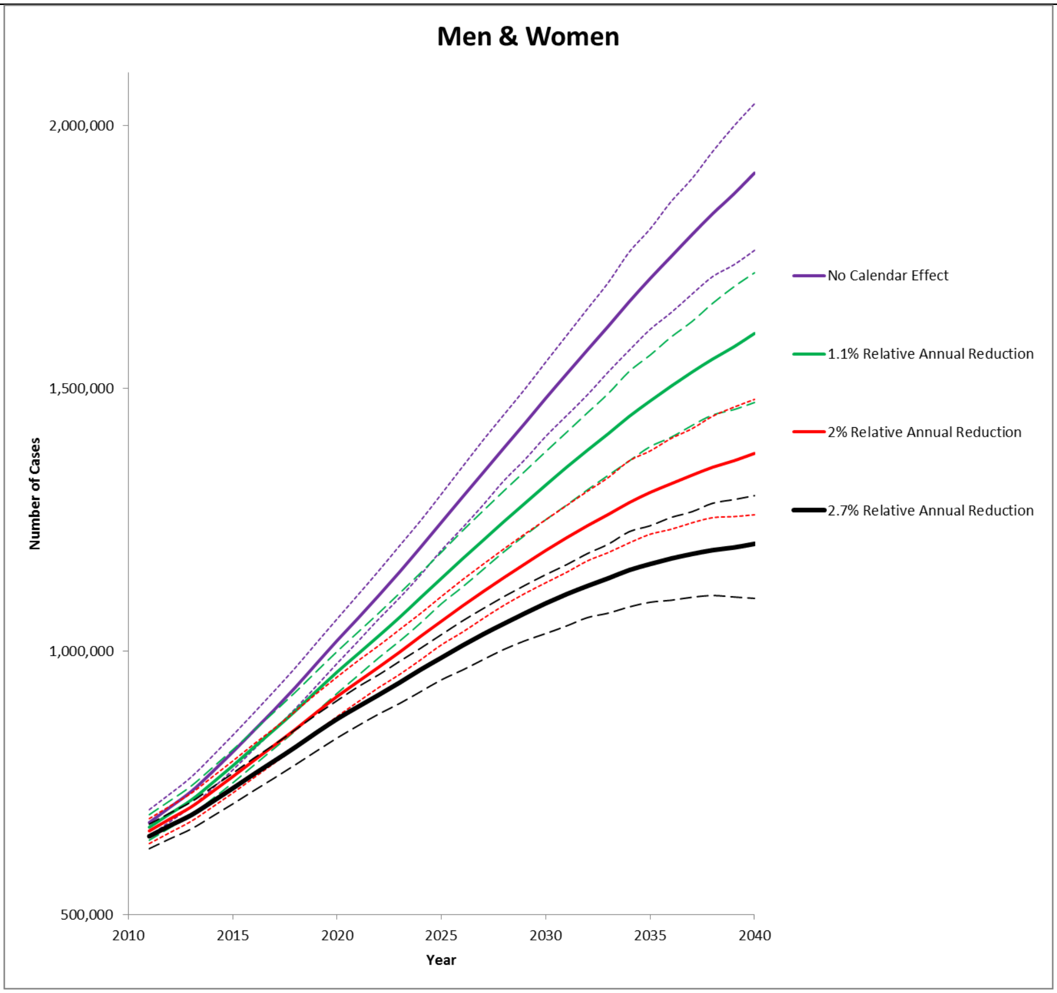

	Assumption: Changes in survival over time are accounted for in the model.	Survival in IMPACT-BAM is indirectly modelled as a function of changing mortality rates. It is assumed that the ratio of mortality rates for each health state in the model compared to the general population is similar to that observed in ELSA and does not change over calendar time. Hence survival with each condition is assumed to improve parallel to improvement in survival at the general population level.

	Assumption: Net effect of changes in risk factors over time would results in the continuation of calendar trends in mortality rates and incidence of dementia and cardiovascular disease observed over the past two decades to the future.	Population levels of risk factors affecting incidence of cardiovascular disease or dementia such as diabetes, smoking, diet, and physical activity, have changed over time. The net effect of changes in risk factors on changes in mortality rates and incidence of cardiovascular disease and dementia have resulted in steady and linear calendar trends as shown above. It is reasonable to assume the likely scenario would be that the net effect of changes in risk factors would be the continuation of observed trends over the past two decades to the future. This analysis forms the baseline modelling scenario. Changes in risk factors and the impact of public health policies and interventions will be explicitly modelled in detail and compared with the baseline scenario. Results of such analysis are extensive and beyond the scope of a single paper.

Note this table mainly reproduced from Ahmadi-Abhari et al., 2017 table 1.[3]

Validation of the model

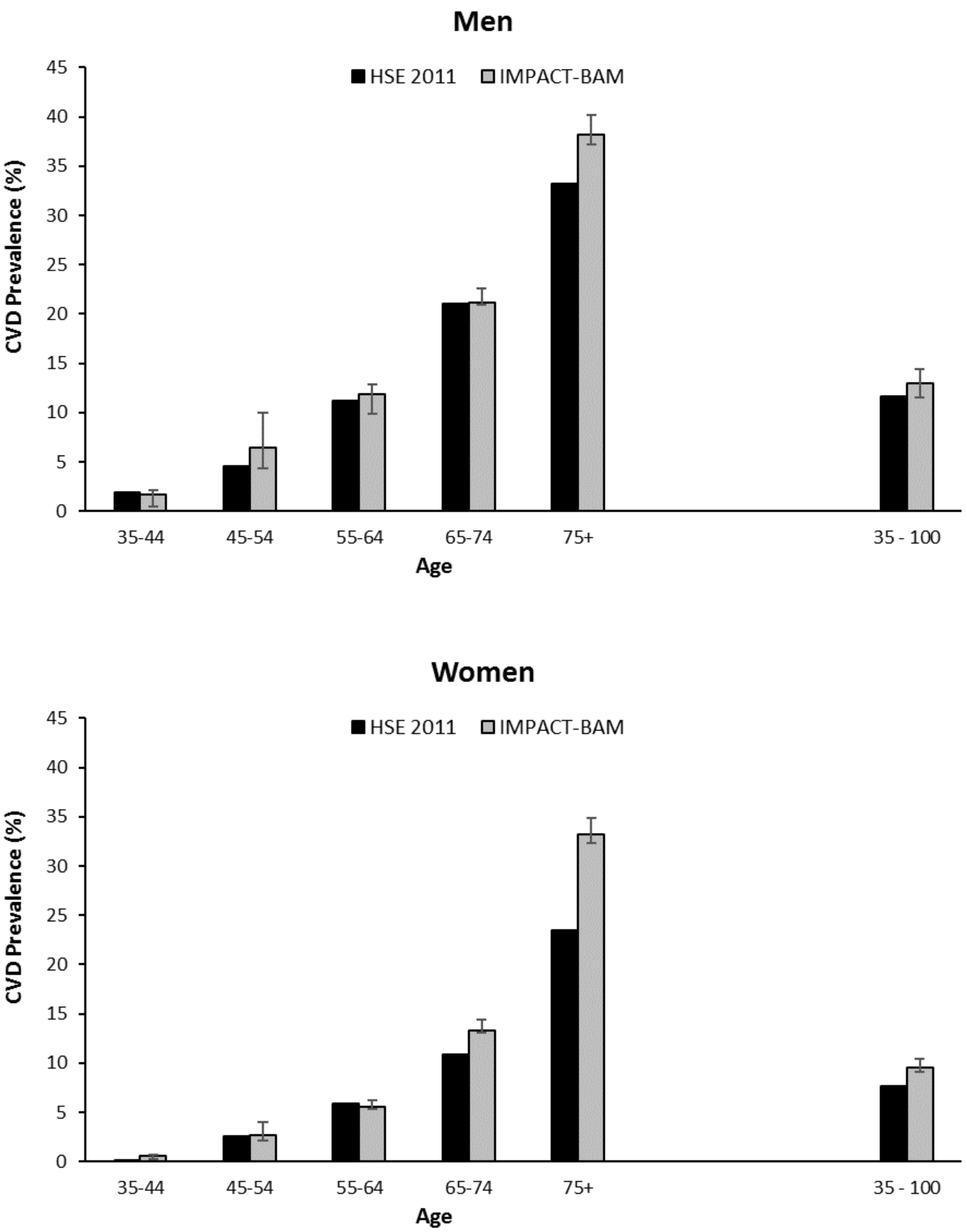

Figure S2 IMPACT-BAM predicted cardiovascular disease prevalence compared with observed estimates from the Health Survey for England in 2011

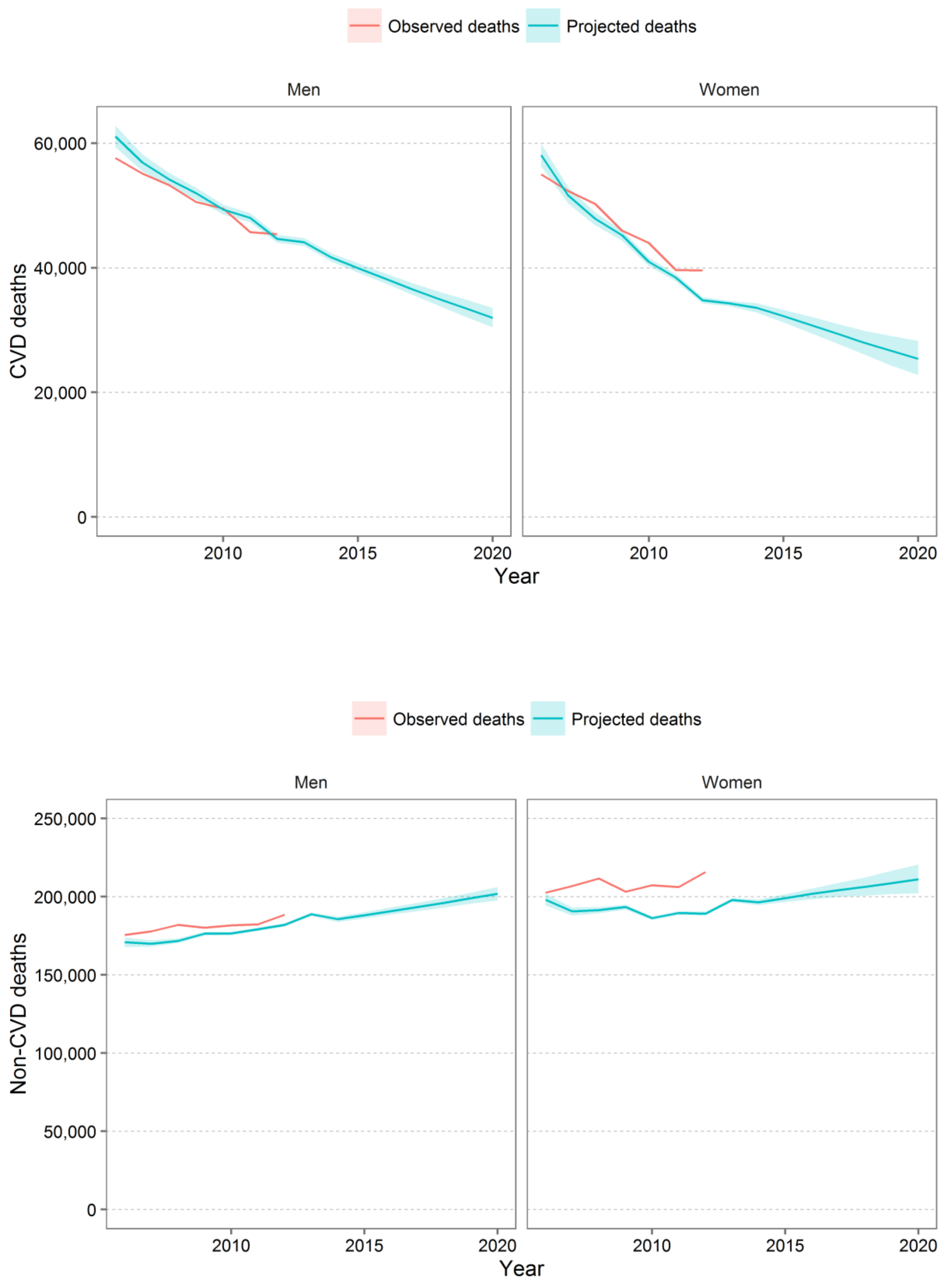

Figure S3 IMPACT-BAM predicted mortality compared with observed estimates from the UK Office for National Statistics

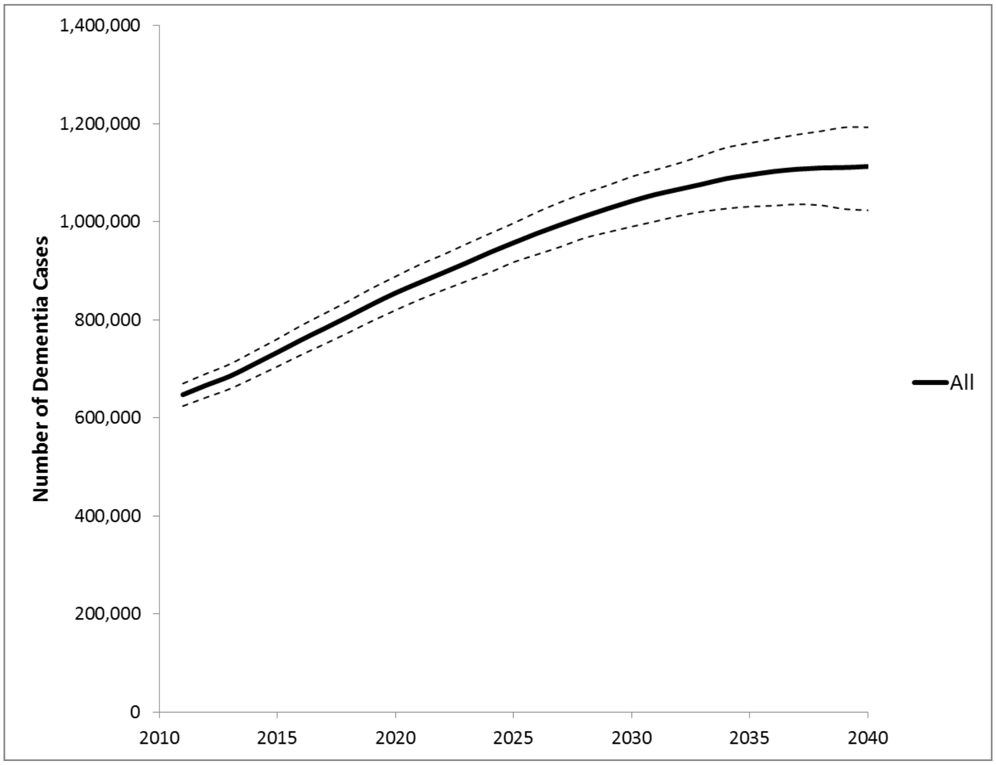

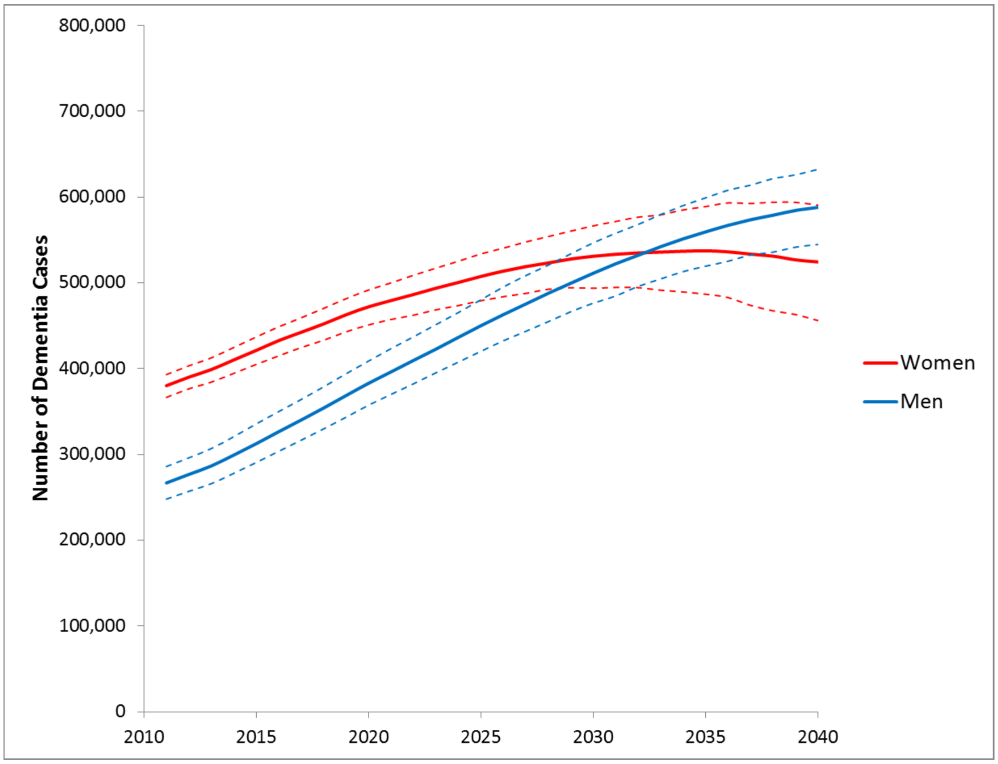

Figure S4 Sensitivity analysis for numbers of cases of dementia assuming cardiovascular incidence does not decline after 2014

Future trends in hypertension prevalence

We considered a continuing downward trend of observed trends in hypertension prevalence 2003-17 as the baseline scenario. Blood pressure is a multifaceted trait, affected by nutrition, environment, and behaviour throughout the life course. The declining trend in hypertension prevalence in high-income countries has declined since 1975 despite increasing body-mass-index.[4,5] This implies that hypertension's other determinants must have improved, and it is reasonable to assume that those nutritional, environmental and behavioural determinants will continue to improve.

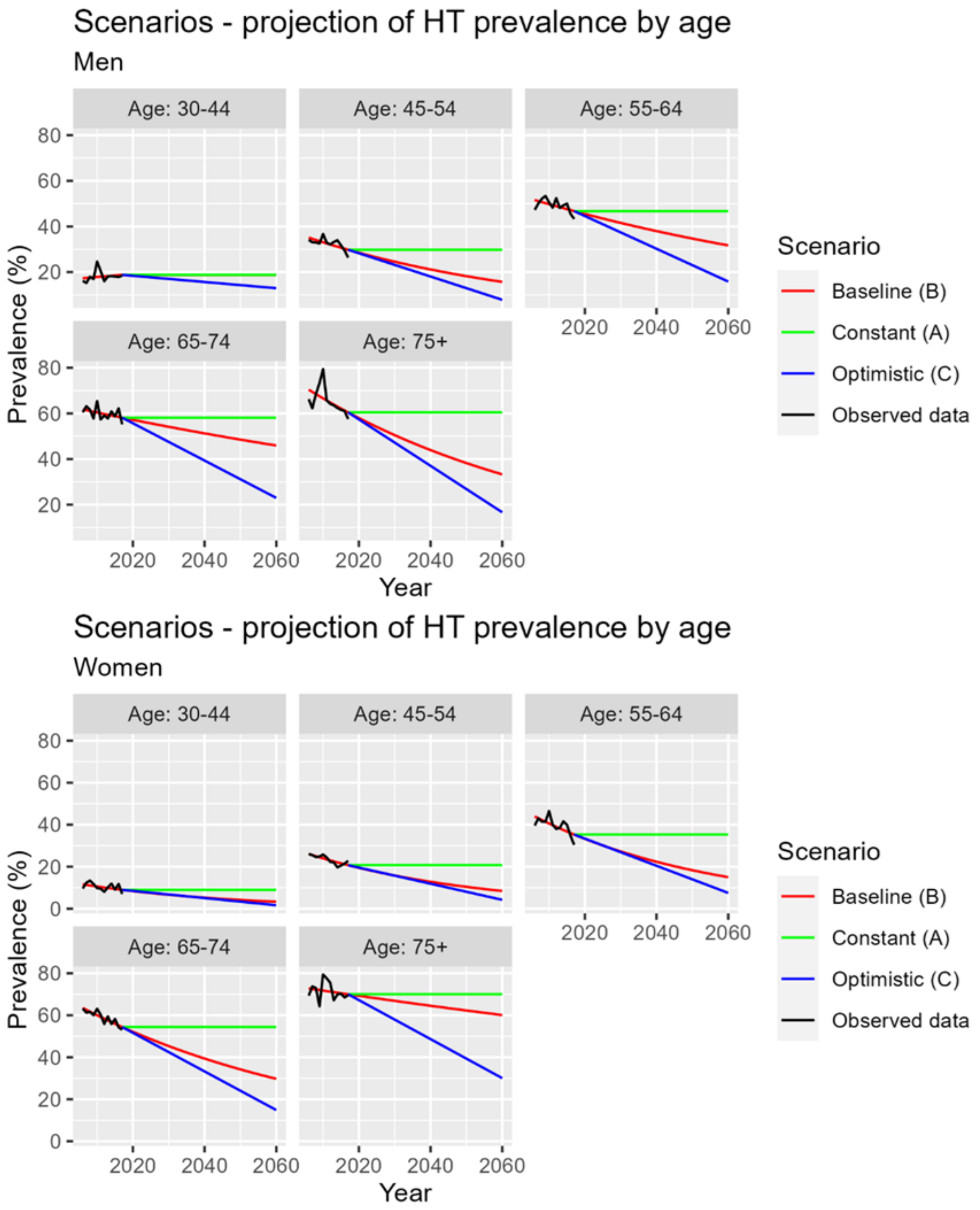

Figure S5 Observed and projected trends in hypertension prevalence in English population by age and sex under the three scenarios; Note that for the visualisation, prevalence proportions are by 10-year age group

Mortality projections

For the present study, projections to 2060 were conducted separately for CVD and non-CVD mortality rates based on observed data reported by World Health Organization mortality data. Based on observed mortality data for 2007-2016, we projected trends for mortality rates in each 5-year age by sex group using Poisson regression assuming a log-linear association between calendar year and mortality. Considering the observed stalling progress in life expectancy in UK after 2010, projection based on the 7 most recent years: 2010-2016 was also presented and compared (Figure S6).

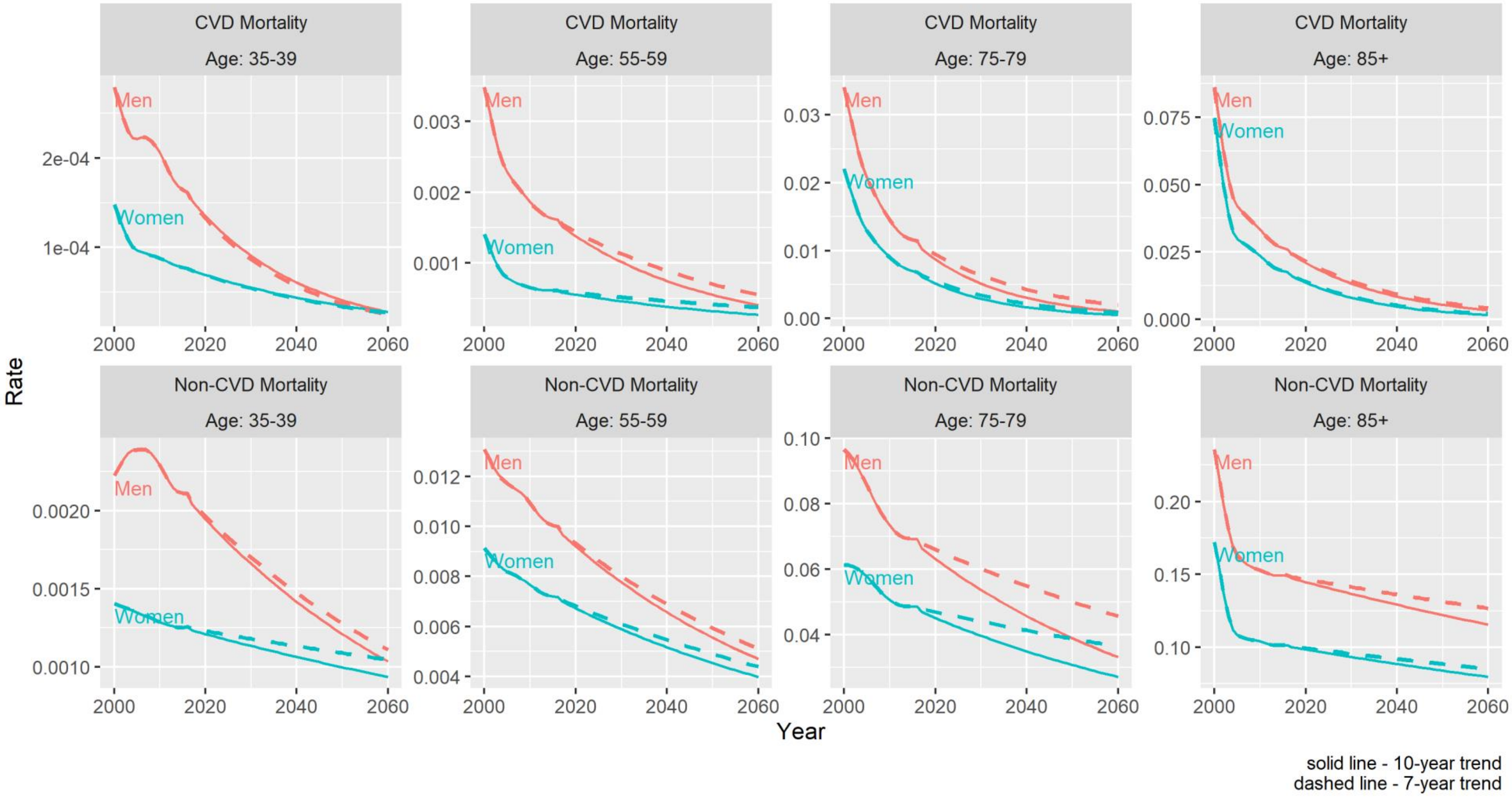

Figure S6 Observed and Predicted CVD mortality in England & Wales estimates using WHO mortality data

Literature review

PubMed was searched using the following search strategy to identify studies reporting the association between blood pressure and the incidence of dementia or incidence of recovery from functional impairment:

Dementia:

blood pressure[Title/Abstract]

AND (**cognitive impairment**[Title/Abstract] OR **mild cognitive impairment**[Title/Abstract] OR **dementia**[Title/Abstract] OR **Alzheimer's disease**[Title/Abstract])

AND (**cohort**[Title/Abstract] OR **longitudinal**[Title/Abstract] OR **prospective**[Title/Abstract] OR **follow up**[Title/Abstract] OR **clinical trial** [Title/Abstract])

AND (**risk**[Title/Abstract] OR **incidence**[Title/Abstract] OR **ratio**[Title/Abstract])

AND **Humans**[Mesh] AND **English**[lang]

Functional Impairment:

blood pressure[Title/Abstract]

AND (**functional impairment**[Title/Abstract] OR **basic activities of daily living**[Title/Abstract] OR **activities of daily living**[Title/Abstract] OR **ADL**[Title/Abstract] OR **disability**[Title/Abstract] OR **frailty**[Title/Abstract])

AND (**cohort**[Title/Abstract] OR **longitudinal**[Title/Abstract] OR **prospective**[Title/Abstract] OR **follow up**[Title/Abstract] OR **clinical trial** [Title/Abstract])

AND (**risk**[Title/Abstract] OR **incidence**[Title/Abstract] OR **ratio**[Title/Abstract] OR **recovery** [Title/Abstract])

AND **Humans**[Mesh] AND **English**[lang]

The following table (Table S1) reports the relative risks (RRs) from our literature review used in the population attributable risk fraction (PARF) approach.

Table S5 Transition probabilities affected by the change in hypertension prevalence

Risk	TPs affected	RRs* from literature search
CVD mortality	P1_9<br>P2_9<br>P3_9<br>P4_9<br>P5_9<br>P6_9<br>P7_9<br>P8_9	2·23 (95%CI, 1·66 to 2·99)[6]
		**Age <60 years**: 2·69 (95% CI, 1·43 to 5·09)[6]
		**Age ≥60 years**: 1·42 (95% CI, 1·10 to 1·83)[6]
Non-CVD mortality	P1_10<br>P2_10<br>P3_10<br>P4_10<br>P5_10<br>P6_10<br>P7_10<br>P8_10	1·62 (95%CI, 1·35 to 1·95)[6]
		**Age <60 years**: 1·57 (95% CI, 1·14 to 2·16)[6]
		**Age ≥60 years**: 1·23 (95% CI, 1·09 to 1·38)[6]
CVD incidence	P1_2<br>P4_3	2·28 (95%CI, 1·87 to 2·78)[7]
		**Age 35-44 years**: 2·65 (95% CI, 1·92 to 3·66)[8]
		**Age 45-59 years:** 2·20 (95% CI, 1·78 to 2·71)[8]
		**Age ≥60 years:** 1·43 (95% CI, 0·97 to 2·12)[8]
Dementia incidence	P1_3, P1_4<br>P2_3	1·49 (95% CI, 1·06 to 2·08)[9]
		**Age 30-50 years**:1·65 (95% CI, 1·14 to 2·38)[10]
		**Age 51-70 years**: 1·28 (95% CI, 1·19 to 1·37)[10]
		**Age 71-90 years:** 0·99 (95%CI, 0·93 to 1·05)[10]
Disability incidence	P2_5<br>P3_6, P1_8,<br>P4_7	1·13 (95%CI, 1·08 to 1·20)[11]
Disability reversal	P5_2, P6_3, P7_4, P8_1	0·68 (95% CI, 0·48 to 0·97)[12]

* RRs for CVD and non-CVD mortality comparing participants with untreated hypertension vs. those without hypertension; RRs for CVD incidence comparing participants with SBP/DBP <120/<80 vs. those with SBP/DBP 140–159/90–99; RRs for dementia incidence per 20 mm Hg higher usual SBP; RRs for disability comparing participants with hypertension vs. those without hypertension.

Effect on life expectancy and compression of morbidity

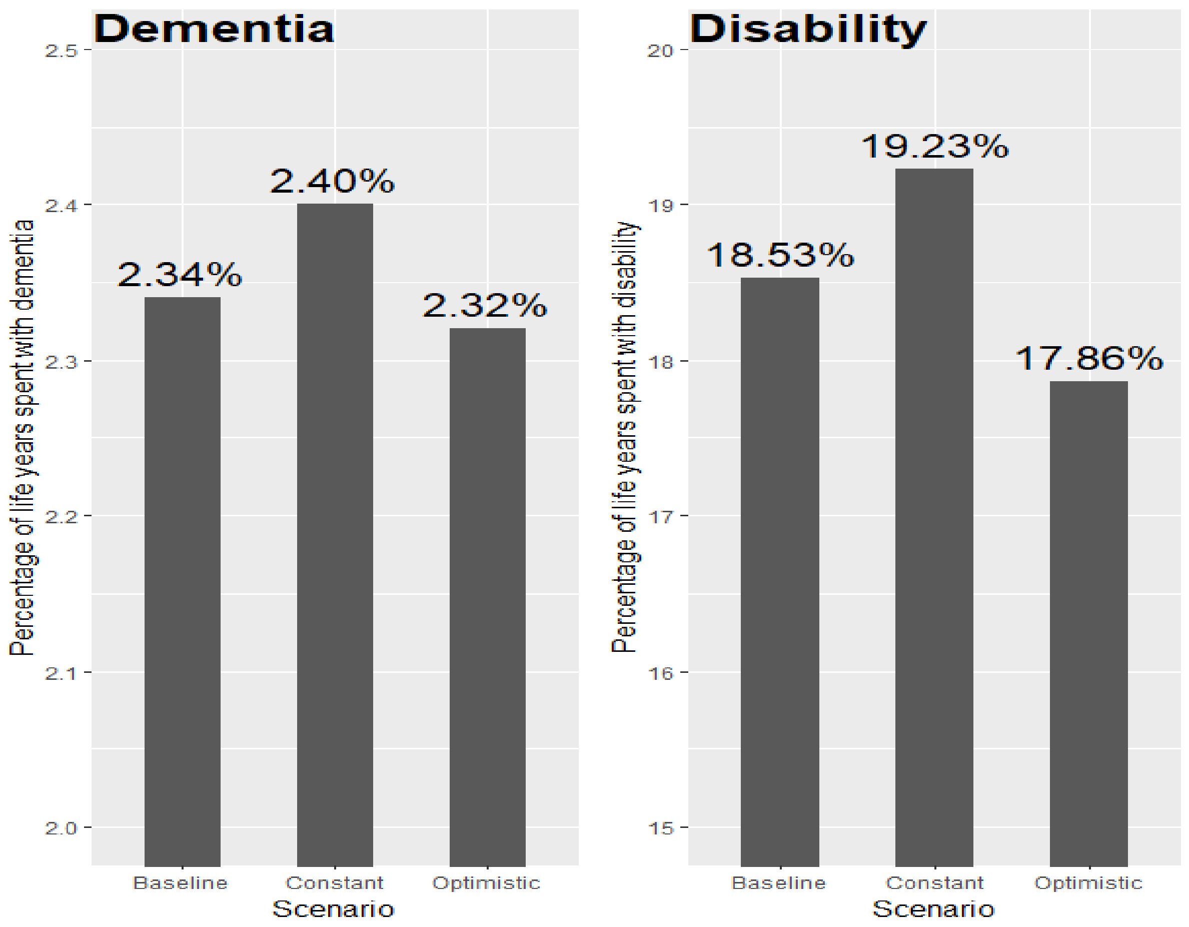

Figure S7 Projected percentage of life years spent with disability and dementia in 2060 for England and Wales, population aged ≥65: three hypertension scenarios

Sensitivity analysis for future mortality trends

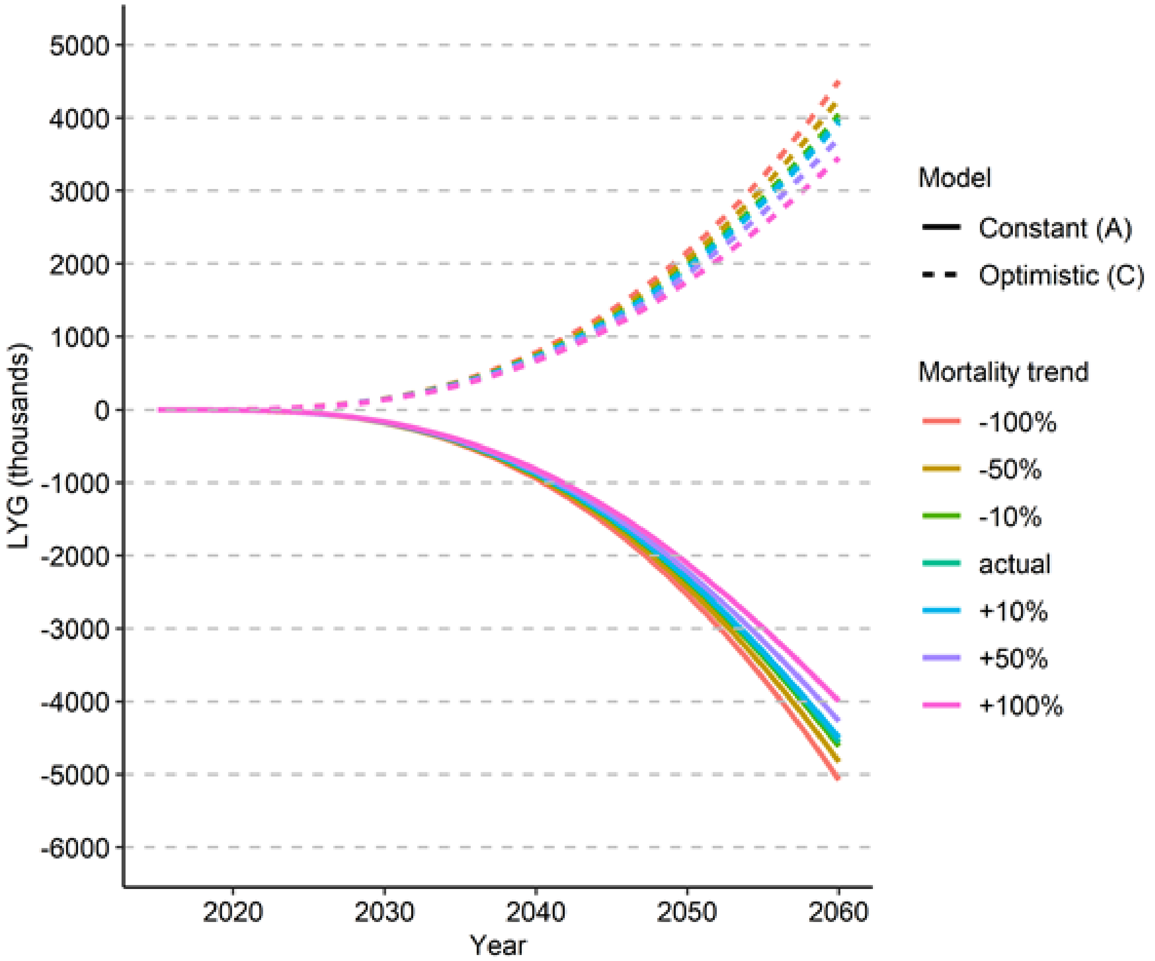

Figure S8 Sensitivity analyses for cumulative number of life years gained (LYG) (thousands) for modelled pessimistic & optimistic scenarios versus baseline scenario for England and Wales, population aged ≥65 over different future mortality trend scenarios (-100%: constant mortality rate since 2016; -50%: 50% of annual decline of mortality that observed in 2010 to 2016; -10%: 90% of annual decline of mortality that observed in 2010 to 2016; actual: continuing the mortality trend in 2010 to 2016; 10%: 1.1 times annual decline of mortality that observed in 2010 to 2016; 50%: 1.5 times annual decline of mortality that observed in 2010 to 2016; 100%: 2 times annual decline of mortality that observed in 2010 to 2016)

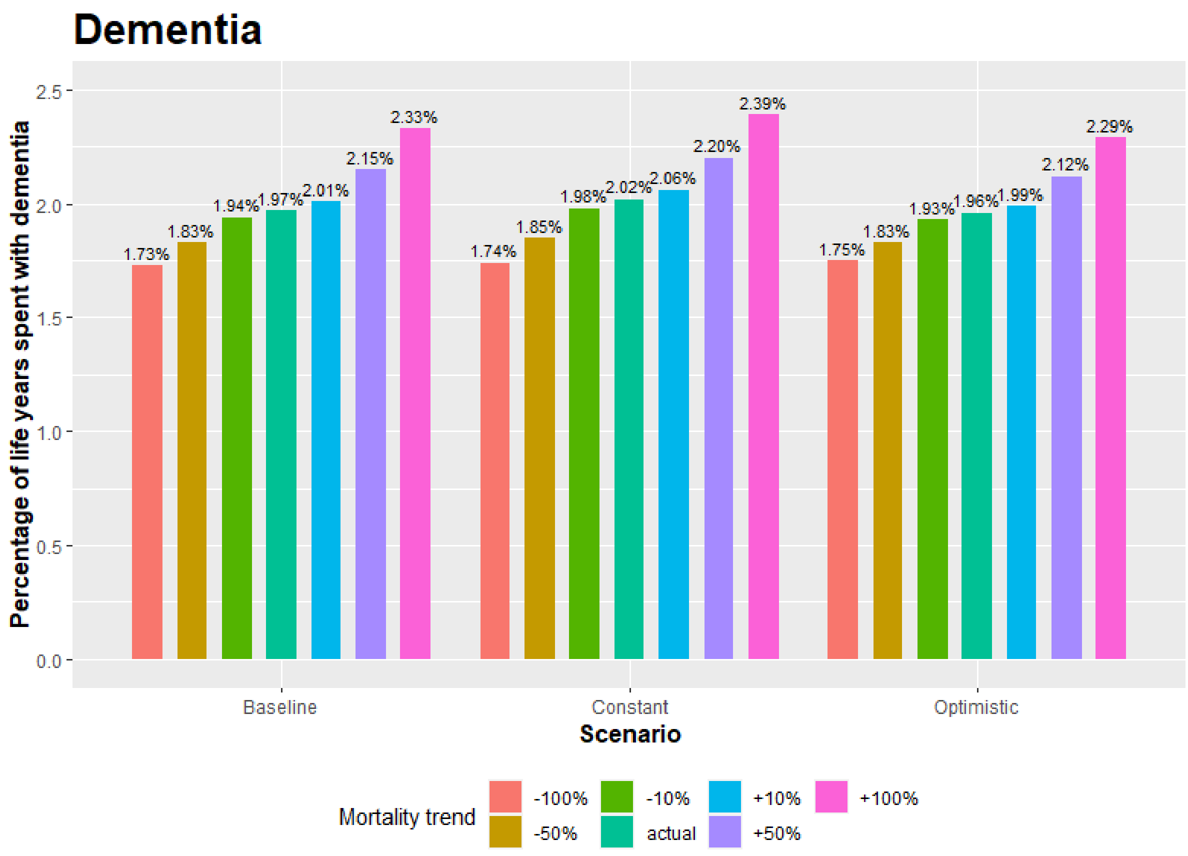

Figure S9 Projected percentage of life years spent with dementia at 2060 for England and Wales, population aged ≥65; Sensitivity analyses over different future mortality trend scenarios (-100%: constant mortality rate since 2016; -50%: 50% of annual decline of mortality that observed in 2010 to 2016; -10%: 90% of annual decline of mortality that observed in 2010 to 2016; actual: continuing the mortality trend in 2010 to 2016; 10%: 1.1 times annual decline of mortality that observed in 2010 to 2016; 50%: 1.5 times annual decline of mortality that observed in 2010 to 2016; 100%: 2 times annual decline of mortality that observed in 2010 to 2016)

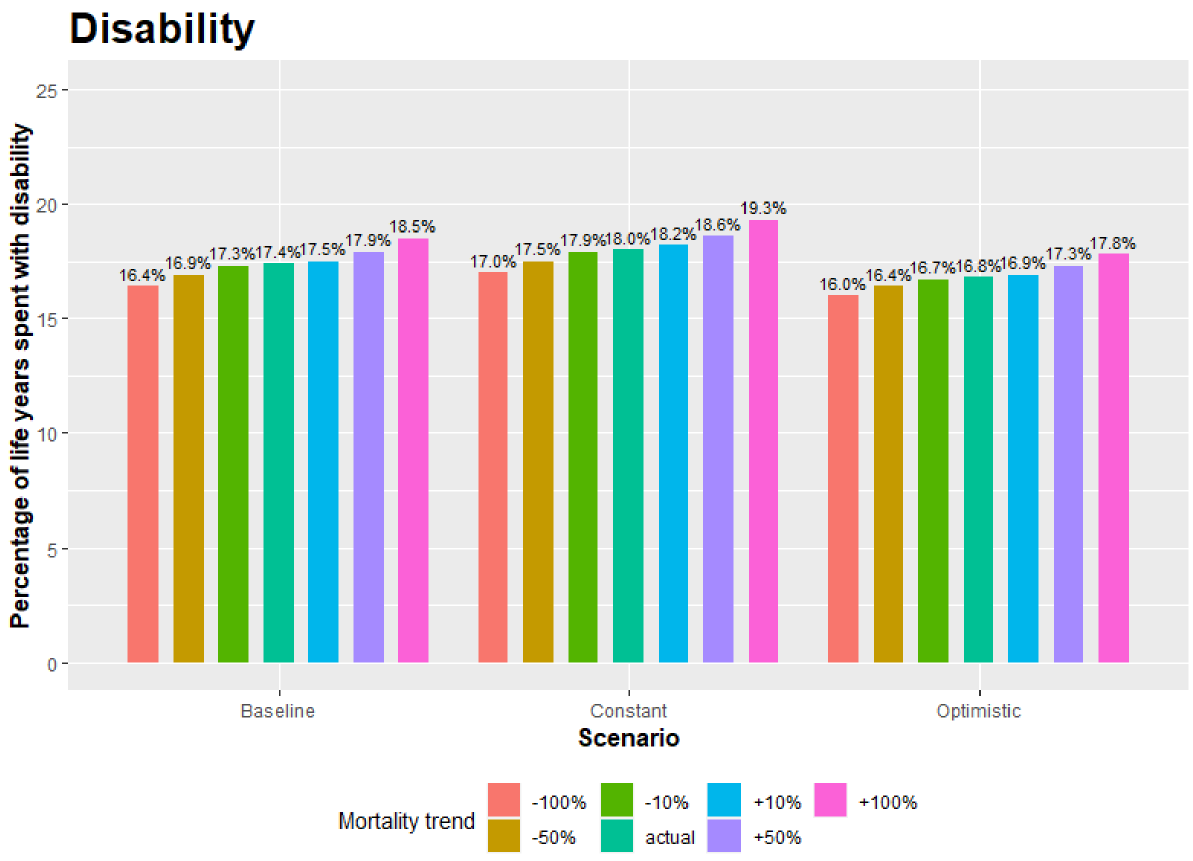

Figure S10 Projected percentage of life years spent with disability at 2060 for England and Wales, population aged ≥65; Sensitivity analyses over different future mortality trend scenarios (-100%: constant mortality rate since 2016; -50%: 50% of annual decline of mortality that observed in 2010 to 2016; -10%: 90% of annual decline of mortality that observed in 2010 to 2016; actual: continuing the mortality trend in 2010 to 2016; 10%: 1.1 times annual decline of mortality that observed in 2010 to 2016; 50%: 1.5 times annual decline of mortality that observed in 2010 to 2016; 100%: 2 times annual decline of mortality that observed in 2010 to 2016)

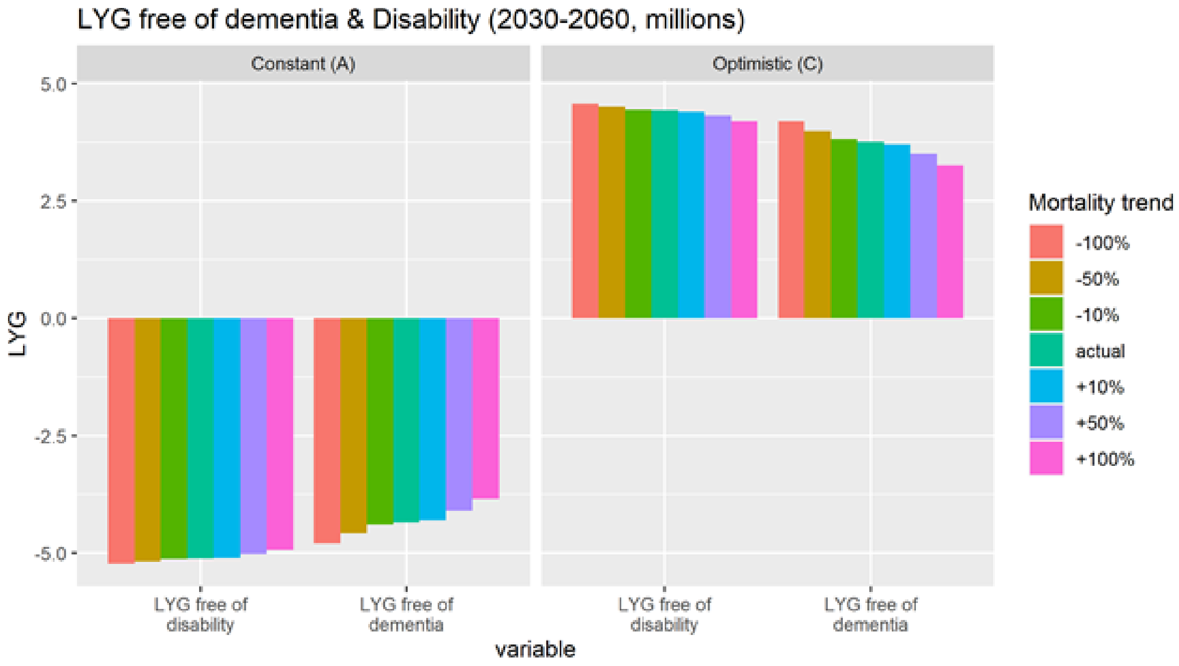

Figure S11 Projected life year gained free of dementia and disability from 2020 to 2060 for England and Wales, population aged ≥65; Sensitivity analyses over different future mortality trend scenarios (-100%: constant mortality rate since 2016; -50%: 50% of annual decline of mortality that observed in 2010 to 2016; -10%: 90% of annual decline of mortality that observed in 2010 to 2016; actual: continuing the mortality trend in 2010 to 2016; 10%: 1.1 times annual decline of mortality that observed in 2010 to 2016; 50%: 1.5 times annual decline of mortality that observed in 2010 to 2016; 100%: 2 times annual decline of mortality that observed in 2010 to 2016)

Reference

1 Guzman-Castillo M, Ahmadi-Abhari S, Bandosz P, *et al.* Forecasted trends in disability and life expectancy in England and Wales up to 2025: a modelling study. *The Lancet Public Health* 2017; **2**: e307–13.

2 Guzman-Castillo M, Gillespie DOS, Allen K, *et al.* Future declines of coronary heart disease mortality in england and wales could counter the burden of population ageing. *PLoS ONE* 2014; **9**: e99482.

3 Ahmadi-Abhari S, Guzman-Castillo M, Bandosz P, *et al.* Temporal trend in dementia incidence since 2002 and projections for prevalence in England and Wales to 2040: modelling study. *BMJ* 2017; **358**: j2856.

4 Zhou B, Bentham J, Cesare MD, *et al.* Worldwide trends in blood pressure from 1975 to 2015: a pooled analysis of 1479 population-based measurement studies with 19·1 million participants. *The Lancet* 2017; **389**: 37–55.

5 Trends in adult body-mass index in 200 countries from 1975 to 2014: a pooled analysis of 1698 population-based measurement studies with 19·2 million participants. *The Lancet* 2016; **387**: 1377–96.

6 Zhou D, Xi B, Zhao M, Wang L, Veeranki SP. Uncontrolled hypertension increases risk of all-cause and cardiovascular disease mortality in US adults: the NHANES III Linked Mortality Study. *Sci Rep* 2018; **8**: 9418.

7 Fagard RH, Cornelissen VA. Incidence of cardiovascular events in white-coat, masked and sustained hypertension versus true normotension: a meta-analysis. *J Hypertens* 2007; **25**: 2193–8.

8 Qi Y, Han X, Zhao D, *et al.* Long-Term Cardiovascular Risk Associated With Stage 1 Hypertension Defined by the 2017 ACC/AHA Hypertension Guideline. *J Am Coll Cardiol* 2018; **72**: 1201–10.

9 Walker KA, Sharrett AR, Wu A, *et al.* Association of Midlife to Late-Life Blood Pressure Patterns With Incident Dementia. *JAMA* 2019; **322**: 535–45.

10 Emdin CA, Rothwell PM, Salimi-Khorshidi G, *et al.* Blood Pressure and Risk of Vascular Dementia: Evidence From a Primary Care Registry and a Cohort Study of Transient Ischemic Attack and Stroke. *Stroke* 2016; **47**: 1429–35.

11 Stevens A, Courtney-Long E, Gillespie C, Armour BS. Hypertension among US adults by disability status and type, National Health and Nutrition Examination Survey, 2001-2010. *Prev Chronic Dis* 2014; **11**: E139.

12 Wong AKW, Lord SR, Sturnieks DL, Delbaere K, Trollor JN, Close JCT. Angiotensin system-blocking medications are associated with fewer falls over 12 months in community-dwelling older people. *J Am Geriatr Soc* 2013; **61**: 776–81.
